# Perceptual dimensions of wood materials

**DOI:** 10.1167/jov.24.5.12

**Published:** 2024-05-24

**Authors:** Jiří Filip, Jiří Lukavský, Filip Děchtěrenko, Filipp Schmidt, Roland W. Fleming

**Affiliations:** 1 The Czech Academy of Sciences, Institute of Information Theory and Automation, Prague, Czech Republic; 2 The Czech Academy of Sciences, Institute of Psychology, Prague, Czech Republic; 3 Experimental Psychology, Justus Liebig University Giessen Germany, Giessen, Germany; 4 Center for Mind, Brain and Behavior, Universities of Marburg, Giessen, and Darmstadt, Germany

**Keywords:** texture, surface, color, categorization, similarity, wood, material, perception, rating, dimension

## Abstract

Materials exhibit an extraordinary range of visual appearances. Characterizing and quantifying appearance is important not only for basic research on perceptual mechanisms but also for computer graphics and a wide range of industrial applications. Although methods exist for capturing and representing the optical properties of materials and how they vary across surfaces (Haindl & Filip, 2013), the representations are typically very high-dimensional, and how these representations relate to subjective perceptual impressions of material appearance remains poorly understood. Here, we used a data-driven approach to characterizing the perceived appearance characteristics of 30 samples of wood veneer using a “visual fingerprint” that describes each sample as a multidimensional feature vector, with each dimension capturing a different aspect of the appearance. Fifty-six crowd-sourced participants viewed triplets of movies depicting different wood samples as the sample rotated. Their task was to report which of the two match samples was subjectively most similar to the test sample. In another online experiment, 45 participants rated 10 wood-related appearance characteristics for each of the samples. The results reveal a consistent embedding of the samples across both experiments and a set of nine perceptual dimensions capturing aspects including the roughness, directionality, and spatial scale of the surface patterns. We also showed that a weighted linear combination of 11 image statistics, inspired by the rating characteristics, predicts perceptual dimensions well.

## Introduction

The visual appearance of materials results from a wide range of physical phenomena including the surface's spectral and angular reflectance characteristics, subsurface light scattering, and spatial variations in pigmentation and surface relief. How the visual system estimates such characteristics remains poorly understood (Anderson, 2011; Bracci & Op de Beeck, 2023), and it also remains unclear which perceptual dimensions the visual system uses to describe and compare different materials (Fleming, 2017).

Capturing a comprehensive representation of a surface's physical appearance requires observing it under a sufficient range of illumination and viewing geometries. Complex photorealistic appearances can be approximated by advanced image-based representations used in computer graphics such as the spatially varying bidirectional reflectance distribution function (Nicodemus, Richmond, Hsia, Ginsburg, & Limperis, 1977) or bidirectional texture function (BTF; Dana, van Ginneken, Nayar, & Koenderink, 1999). However, these representations are extremely high-dimensional, and there is no straightforward mapping between such representations and subjective visual appearance characteristics. Somehow, the visual system summarizes the overall “look” of complex, spatially-varying appearances to compare and contrast different materials. Everyday experience suggests that observers do not need to view a material from all possible view- and lighting-directions to obtain a distinct impression of its appearance. Yet, although the perceptual representation of materials is surely lower-dimensional than a complete physical description of the surface, there are nevertheless many potential dimensions that the visual system might draw on to describe materials (e.g., overall albedo, relief, glossiness, contrast of surface patterns).

Our goal here was to establish and evaluate approaches for identifying key visual properties of materials—so-called “perceptual dimensions,” from human observations of a dynamic video of a flat wooden material sample. We use the terms “perceptual dimension” and “perceptual embedding” to refer to subjective visual representations of material appearance. Different perceptual dimensions refer to the different visual aspects or attributes that contribute to the overall appearance of the visual input (e.g., color, texture, orientation). Therefore they can help in understanding the key image cues and physical factors that contribute to material appearance. Together, the perceptual dimensions form a perceptual embedding that sensory data into a continuous mathematical space which captures the inherent relationships between different materials, allowing for more efficient processing and analysis.

We still do not understand much about the perceptual dimensions and how they contribute to observers’ judgments of appearance. Which characteristics do observers use to compare different materials? Is there a “ranking” of characteristics, such that some aspects of appearance dominate comparisons between materials, whereas others play a secondary role? How specific are certain characteristics to particular classes of materials? Previous work on material perception has often focused on highly constrained sets of stimuli varying in one or a small number of physical properties (Ferwerda, Pellacini, & Greenberg, 2001; Fleming, Dror, & Adelson, 2003; Fleming, Bülthoff, 2005; Motoyoshi, Nishida, Sharan, & Adelson, 2007; Wendt, Faul, & Mausfeld, 2008; Wendt, Faul, Ekroll, & Mausfeld, 2010; Fleming, Jäkel, & Maloney, 2011; Marlow, Kim, & Anderson, 2012; Paulun, Schmidt, van Assen, & Fleming, 2017; Van Assen, Barla, & Fleming, 2018). Other studies have investigated appearance judgments and categorization based on photographs (e.g., Bell, Upchurch, Snavely, & Bala, 2015; Fleming, Wiebel, & Gegenfurtner 2013; Sharan, Rosenholtz, & Adelson, 2009; Sharan, Liu, Rosenholtz, & Adelson, 2013; Sharan, Rosenholtz, & Adelson, 2014; Wiebel, Valsecchi, & Gegenfurtner, 2013). However, in most cases, it is the experimenters that define which characteristics are judged by participants.

**Figure 1.**
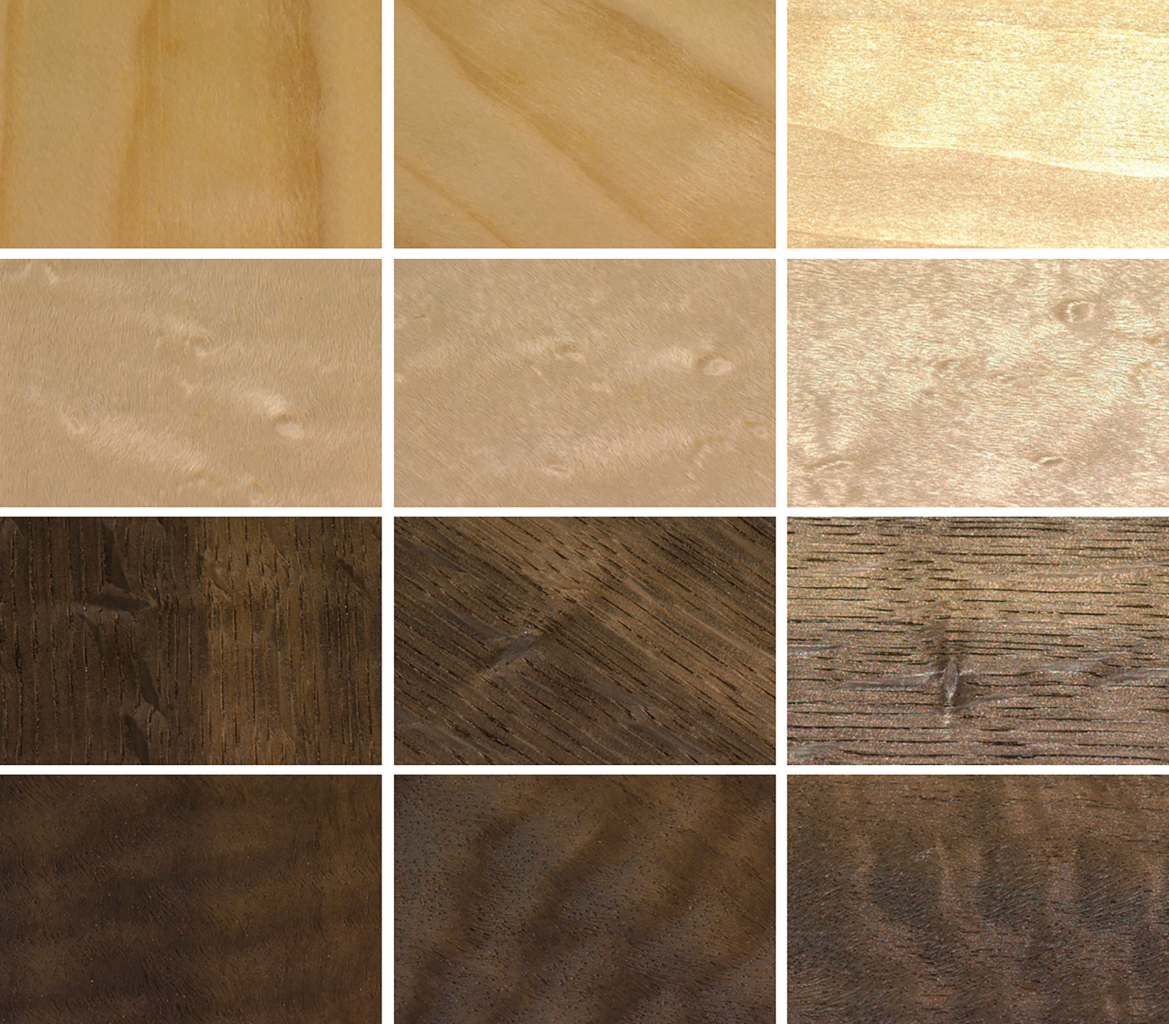
An example of the effect of viewing angle on the appearance of four wood samples (shown in rows).

Here we combined this tradition with a more data-driven approach in order to identify dimensions underlying appearance judgments for a set of thirty samples of planar wood veneer with distinctive surface patterns and textures. Wood is a challenging material to characterize due to its complex and varied appearance. It is associated with decorative attributes and is widely used for furniture and interior design. Its structure consists of elongated cells, which are radially oriented rays and longitudinal cells or vessels forming growth rings (Lewin & Goldstein, 1991). Hardwoods tend to have a tighter grain pattern compared to softwoods, resulting in various levels of texture, color, smoothness, grain density, and straightness. All these aspects are impacted by sawing direction and the sample location in the tree trunk. The final visual structure is given by an intersection of a sawing plane with three-dimensional wood structure. Wood has high natural variability in aesthetic characteristics among different species and surface treatments. Previous studies have shown that patterns of anisotropy, color variations and gloss are the major factors influencing the visual (Nakamura, Masuda, & Shinohara, 1999; Wan, Li, Zhang, Song, & Ke, 2021), multimodal (Fujisaki, Tokita, & Kariya, 2015) aesthetic appeal of wood with impacts on people's preferences (Manuel, Leonhart, Broman, & Becker, 2015), and emotions related to wooden surfaces (Nordvik, Schütte, & Broman, 2009). To the best of our knowledge, all previous studies of wood appearance relied on static stimuli to derive subjective ratings of predefined attributes or their relationship to physical attributes of wood surfaces. This ignores how variable the appearance of even a single sample can be across changes in viewpoint relative to the surface and lighting, as it is shown in Figure 1.

Our contribution to the prior work is twofold. First, our work uses dynamic (rotating) rather than static stimuli, showing the appearance of the 30 wood samples across variable lighting and viewing conditions. This allowed participants in our experiments to take into account the look of the surface both with and without specular reflections. The appearance of wood can change substantially as a function of lighting angle, especially because the elongated nature of fibers and wood grain can lead to anisotropic reflectance characteristics. This makes wood a particularly rich class of materials to consider. To give participants a reasonable summary of the range of appearances exhibited by each sample, we used movies of the samples rotating. The wood samples were carefully selected from a base set of several hundred samples to deliver as much visual variability as possible.

Second, instead of relying solely on a possibly incomplete list of predefined visual attributes, we also used similarity judgements to identify the core dimensions underlying judgments of wood. Similarity judgements are an established method for characterizing the multidimensional space underlying mental representations, previously used to understand perceptual dimensions in object categories (Hebart, Zheng, Pereira, & Baker, 2020), materials (Schmidt, Hebart, & Fleming, 2022) or scenes (Josephs, Hebart, & Konkle, 2023). In contrast to the previous studies, we search for perceptual dimensions underlying similarity judgments within a single category.

**Figure 2.**
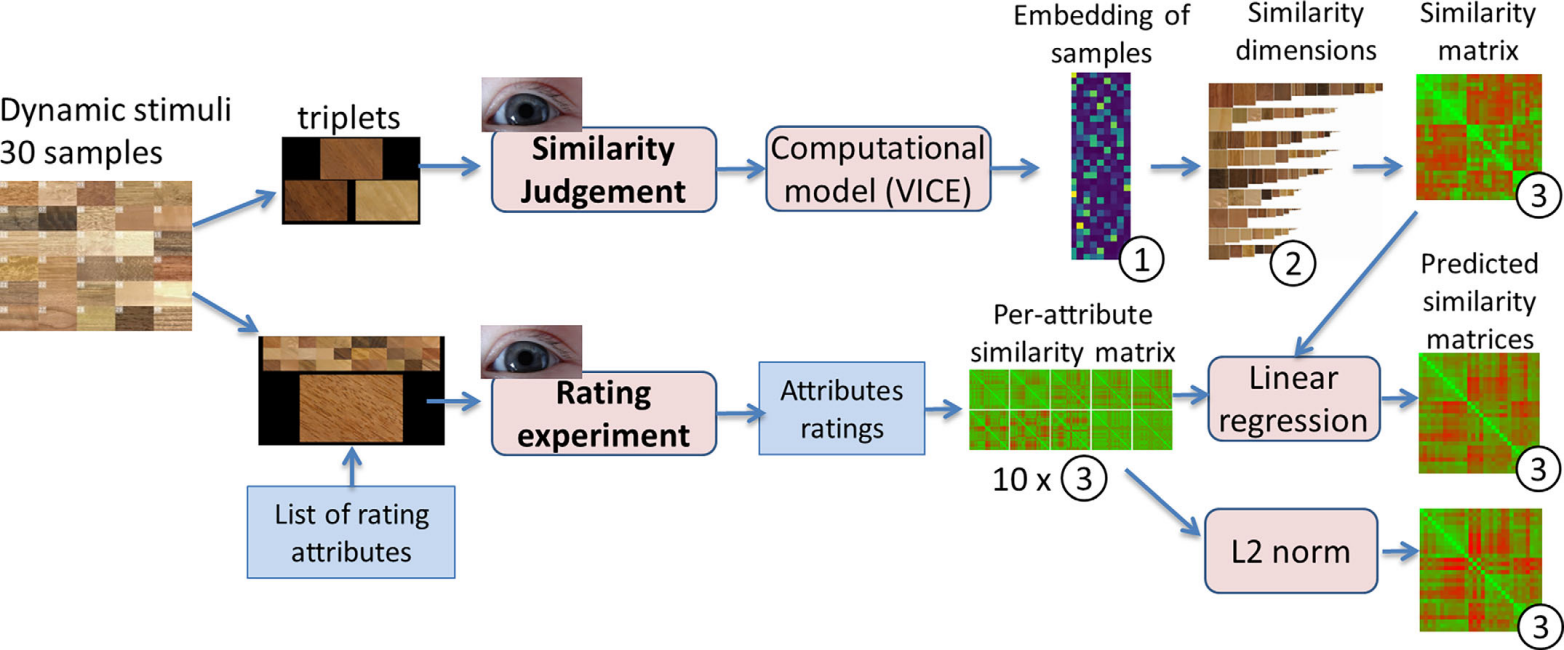
An overview of the two psychophysical experiments on dynamic stimuli of 30 wood samples, along with their respective analysis pipelines. Details of data visualizations 1–3: (1) Embedding of 30 samples (rows) into n similarity dimensions derived from the VICE algorithm (columns). (2) Ordering of the 30 samples (columns) along n similarity dimensions (rows) according to their embedding values. (3) Similarity matrix of 30 × 30 samples (green values indicate high similarity).

Specifically, we sought to derive a relatively small number of perceptual dimensions that capture judgments of similarity between movies of the samples. It is important to note that for a given similarity judgement, only a subset of all dimensions might come into play. Here, we sought to find a set of dimensions that together capture the similarity relationships between multiple subsets of materials from our sample of thirty wooden materials. In order to do this, we first crowd-sourced 1218 perceptual similarity judgments from 56 participants as shown in the upper part of the block scheme in Figure 2. We then applied an analysis method based on sparse, non-negative matrix factorization (Variational Interpretable Concept Embeddings; Muttenthaler et al., 2022) to infer a set of dimensions that can predict the similarity judgments. We show that even with a small dataset of thirty samples, the method was able to derive perceptual dimensions that predict the similarity judgments. Specifically, our model identified nine dimensions that together could explain over 75% of the variance in the similarity judgments. Additionally, we showed that a linear regression of standard image statistics obtained from stimuli videos predicted the majority of the perceptual dimensions well.

In addition to the similarity judgments, we also asked a set of 45 participants to judge ten experimenter-defined appearance characteristics for each of the samples (brightness, glossiness, colorfulness, directionality, complexity, contrast, roughness, patchiness/regularity, line elongation, spatial scale) as shown in the bottom part of the block scheme in Figure 2. The purpose of this was twofold. First, we sought to use the values of these interpretable rating scales to facilitate interpretation of the dimensions derived from the similarity judgments. Second, we sought to cross-validate the embedding of the samples within the 9D space. We reasoned that if different samples are represented in a multidimensional perceptual similarity space—with similar samples close to one another and dissimilar ones further apart—then it should be possible to probe this space through multiple complementary methods (i.e., similarity judgments and subjective feature ratings).

Our main contributions are the following:

• We learned that the perception of wood material appearance is challenging and can be represented by five to 10 perceptual dimensions.
• We identified particular features of wood materials that participants use when making similarity judgements, related mainly to roughness, directionality and scale of visual features.
• We tested two methods for creating a mapping that describes the relationship between material stimuli: similarity judgments and attribute ratings. Our results demonstrate that the two methods yield embeddings that are substantially correlated but not identical (sharing approximately 50% variance).
• Our analysis shows a significant proportion of shared variance between individual ratings and selected similarity dimensions.

## Experiment 1

In the first experiment, we collected sparse similarity judgements and used machine learning to infer the full pairwise similarity matrix and to test the embedding of samples in the latent space of wood appearance.

### Methods

#### Participants

Fifty-six participants were recruited using the online crowdsourcing platform Prolific (mean age = 40.5, SD = 16.6, 35 males). All participants reported normal or corrected-to-normal vision and no color vision impairments; we did not perform any color vision and visual acuity checks. On average, the experiment took 14.0 minutes (SD = 4.9). The participants were reimbursed with 2.1 GBP. All studies within this article were approved by the Ethics Board of the Institute of Psychology, Academy of Sciences of the Czech Republic (PSU-308/Brno/2022).

#### Apparatus and stimuli

**Figure 3.**
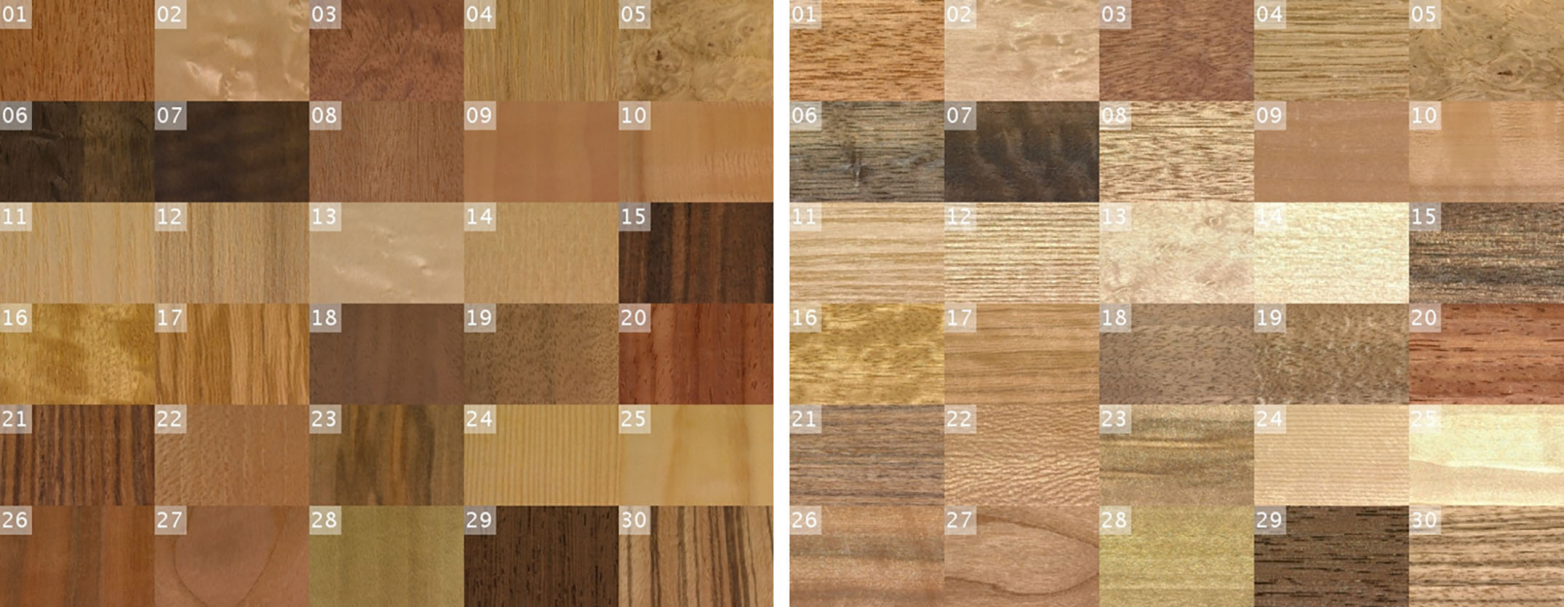
All 30 samples of wood veneer for (left) specular, (right) non-specular (90° rotated) view/light geometries.

We used 30 flat standard non-coated wood veneer samples that are used for furniture manufacturing (wood species are listed in Table 1). We captured video sequences of slow rotations of the samples. Figure 3 shows the initial (left) and final (right) frames of each video sequence, capturing specular and non-specular view/light geometries for all samples. Video samples and additional materials are available at https://osf.io/tz245.

**Table 1.** A complete list of wood species used in the experiment.

	
01	Afzelia
02	Masur birch
03	Pommele bubinga
04	Oak
05	Burr oak
06	Smoked oak
07	Eucalyptus
08	Gaboon
09	Pear
10	European apple
11	White ash
12	Ash heartwood
13	Maple burl
14	European lime (linden)
15	Macassar ebony
16	Movingui (lemon)
17	Olive
18	European walnut
19	Peruvian walnut
20	Padauk
21	Rosewood
22	Plane
23	Satinwood
24	Spruce
25	Spruce knotted
26	Tineo
27	American cherry
28	Tulipwood
29	Wenge
30	Zebrawood

All images in the video sequences were 42 × 42 mm areas of the samples, captured by the UTIA goniometer (Filip et al., 2013). In accordance with industry standards in material observation (McCamy, 1996), we fixed the polar angle of the camera and light to 45° and only varied azimuthal angles to allow for faster measurements. Each sequence starts with a difference of 90° between the azimuthal angles of light and camera and includes a movement of the camera by 90° (arriving at a difference of 180° between azimuthal angles), resulting in specular and nonspecular material behavior as shown in Figure 3. Each four-second sequence consists of 60 image frames, repeated in reverse order to create a continuous loop of rotating material. See Supplementary Video S1.

**Figure 4.**
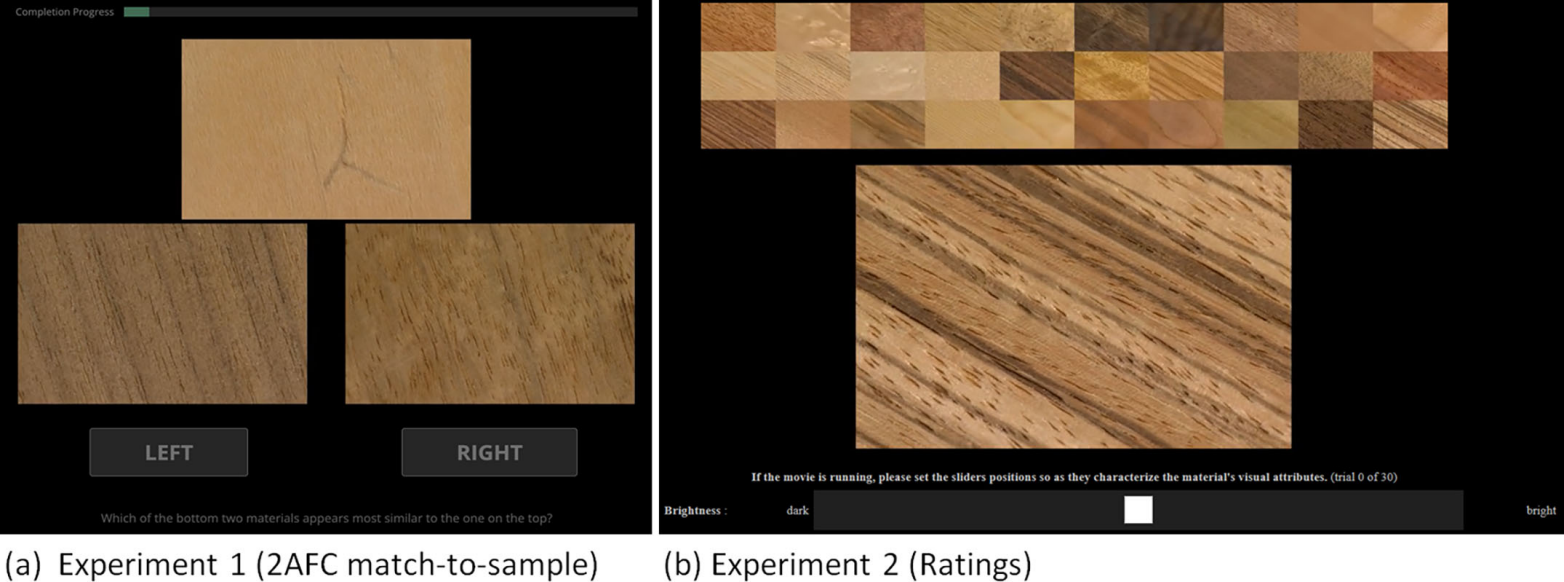
Example of stimuli frames of (**a**) the similarity judgement experiment, where participants responded to: “Which of the bottom two materials appears most similar to the one on the top?” and (**b**) the rating experiment, where participants rated individual samples according to different visual attributes.

To allow for smooth presentation in the experiment, the image frames of all samples were cropped and downsampled to 400 × 260 pixels, and combined into single-trial frames with three samples on a black background at qHD (quarter high definition, 960 × 540 pixels) as shown in Figure 4(a). Each sequence was started at a random time of the continuous loop to prevent participants from responding to the initial frames of the video sequence.

Because data was collected online, we did not control for viewing distance (viewing angles) or monitor settings. However, a post-hoc analysis of monitor settings showed a minimal screen resolution of 980 x 577 pixels, which allows for a full-resolution presentation of our stimuli.

#### Experimental procedure

Experiment 1 consisted of 93 trials. In each trial, participants judged the similarity of three presented samples as shown in Figure 4(a), by deciding which of two match stimuli (at the bottom of the screen) was more similar to the test stimulus (at the top of the screen; 2AFC match-to-sample design). Because we study similarity within a single material category (wood), we hypothesized a relatively low number of three to five meaningful perceptual dimensions. In line with the recommendations in (Haghiri, Rubisch, Geirhos, Wichmann, & von Luxburg, 2019) (30 samples and 3–5 dimensions: 900–1500 trials), we tested 1218 triplets, selected to account for 10% of all triplets combinations with fixed match stimulus full similarity matrix. We aimed for the most balanced coverage of materials. Specifically, the 1218 triplets were created so that each sample was presented as a match stimulus (the top one) a similar number of times (i.e., 40 times [except for 18 stimuli for which it was 41 times]). Also, for all these 40 (41) positions for a given material, each of the 29 remaining materials was used equally often (two or three times). To compute the noise ceiling and subjects’ consistency, we replicated the same set of triplets four times, whereas two times an order of the test stimuli was swapped, giving a total of 4872 triplet trials.

Across all triplets, each sample was presented as a test stimulus in 160 to 164 trials and as a match stimulus in 304 to 344 trials. Each triplet was judged four times (i.e., by four different participants). Two out of four repetitions swapped the left and right match stimuli to control for a potential response bias. Each participant was presented with one of 28 unique trial sets or its copy with swapped match stimuli.

Data were collected online using a custom script in the jsPsych framework (De Leeuw, 2015). After reading the instructions, participants completed three practice trials and 90 experimental trials (87 trials plus 3 catch trials). They initiated each trial by clicking the “Start” button, after which a video with the three samples started looping (Figure 4a). Participants responded to the instruction below the video (“*Which of the bottom two materials appears most similar to the one on the top?*”) by clicking on the “left” or ”right” button at the bottom. The response stopped the loop and initiated the next trial, with a progress bar at the top showing the number of remaining trials. Catch trials were presented at fixed positions (fortieth, sixty-fifth, and eighty-fourth trials) and featured the same sample presented twice, as standard and match stimulus, yielding a ground truth correct response.

#### Data analysis

All data are available from the following public repository: [https://osf.io/tz245/]. We next sought to identify a set of perceptual dimensions—with values for every sample—that could account for the observed pattern of similarity responses. To do this, we analyzed the responses using Variational Interpretable Concept Embeddings (VICE; Muttenthaler et al., 2022). This algorithm takes as input the raw triplet judgements, derives from these a sparse (i.e., incomplete) similarity matrix, and estimates the full pairwise similarity matrix. In the process, it iteratively estimates a set of underlying dimensions that could account for the observed responses. As participants in our similarity judgement study chose the more similar of two samples to a match sample (i.e., 2AFC task), we applied the target matching variant of VICE (instead of its odd-one-out variant procedure).

Several of the VICE algorithm's hyperparameters can affect its results, including the number of dimensions. To validate the performance of the model, we created random splits of our participants’ similarity judgements into training (90% of responses) and test sets (10% of responses). Then, we performed a limited grid search for selected hyperparameters of the model: learning rate [0.0005, 0.001, 0.002], mixture of distributions in the spike-and-slab prior [Gaussians, Laplace], spike (a prior of probability at zero values) [0.125, 0.25, 0.75], slab (a prior of probability for the non-zero values) [0.2, 0.5, 1.0], and probability of relative weighting of the distributions [0.4, 0.5, 0.6]. The training typically converged within 200 epochs and typically resulted in between eight and 11 dimensions (minimum four, maximum 14 dimensions). We refer to the VICE model dimensions as similarity dimensions in the rest of the text. Details of the model selection and training process are reported in Section 1 of the Supplementary Material. The formula calculating the similarity matrix from observers’ similarity judgements is given in Supplementary Material as Equation (1).

### Results

#### Consistency of similarity judgement responses

Our results show that participants were highly consistent in their similarity judgments. When analyzing interindividual consistency based on the four repetitions of each triplet, in 569 triplets (47%) all four responses were the same, in 439 triplets (36%) three responses were the same, and in 210 triplets (17%) responses were on par. This suggests that in the majority of trials (83%) subjects were consistent, only in the remaining 17% they were at chance. Also, when comparing sequences with their copies with swapped match stimuli, swapping resulted in a different response in only 61 trials (5%).

#### Deriving perceptual dimensions from similarity judgements

Based on the parameter grid search (see Section 1 of the Supplementary Material), we picked the best performing model (nine dimensions; accuracy on the training set = 0.760; accuracy on the test set = 0.769). Importantly, even though the number of similarity dimensions varied between different resulting models from the parameter grid search, the meaning of those dimensions was highly preserved. Specifically, the embeddings obtained from the first five best VICE models (with five to nine dimensions and different hyper-parameters) were highly similar (mean correlation between similarity matrices of the four next best models to that of the best model was *R* = 0.922). See the Supplementary Material for details. Thus, in the following we analyze the best performing VICE model under the justified assumption that it is representative of a family of models with similar embedding.

**Figure 5.**
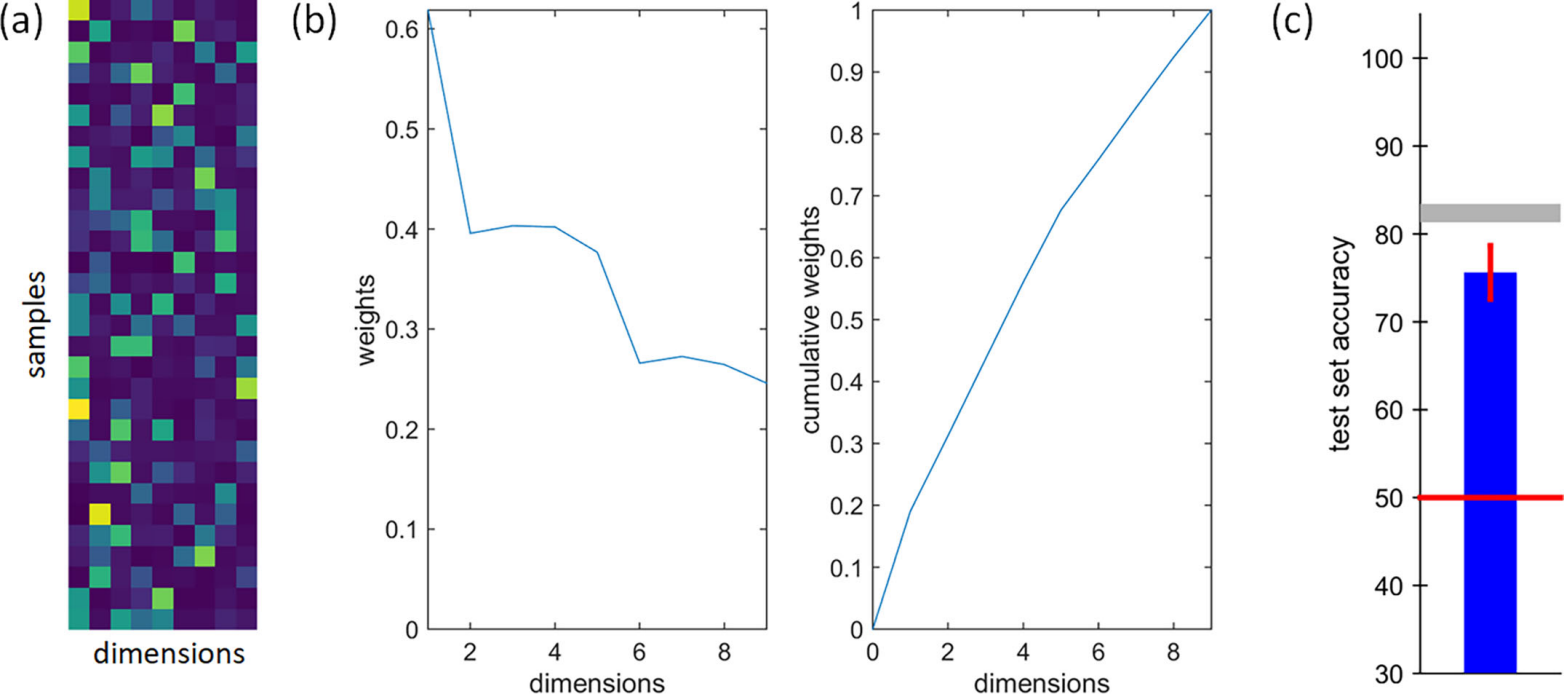
Details on the nine similarity dimensions of the best VICE model: (**a**) estimated embedding, (**b**) sum of embedding values for individual dimensions (columns of a) and their cumulative sum, and (**c**) average accuracy on test set (blue) with 95% confidence interval error-bar (red), approximate noise ceiling (gray), and chance level (red).

The resulting embedding as shown in Figure 5a is quite sparse, with on average only six values > 20% percentile in each similarity dimension. Figure 5b shows the sum of loadings for individual dimensions and suggests that the first five dimensions have a much higher impact than the remaining four. Figure 5c compares how well the similarity responses from participants can be approximated by the values estimated from the VICE model. Chance performance in the 2AFC match-to-sample task (red) is 50%, with the interparticipant noise ceiling (gray) at 82%. Similarly to Hebart et al., 2020, we estimated an approximate noise ceiling as the average agreement across the four repetitions of each triplet. This approximates the best possible prediction any model could achieve for our dataset, given the variation in the judgement data.

**Figure 6.**
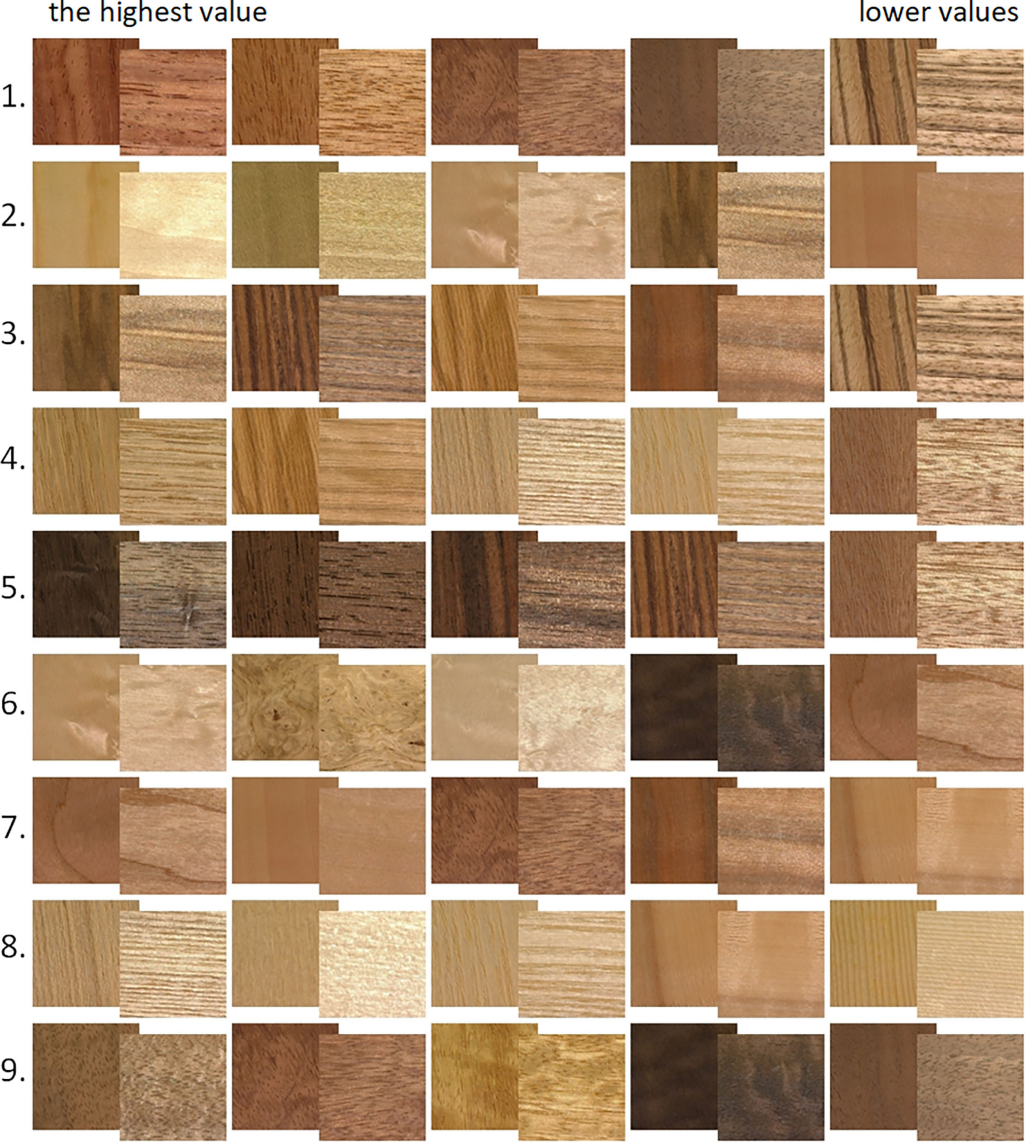
Five samples for each similarity dimension rank-ordered based on embedding values. Each video sample is represented by both the most non-specular and most specular condition. See left side of Supplementary Video S2.

Figure 6 shows samples rank-ordered by their embedding values in each of the nine similarity dimensions (highest values to the left). Each video sample is represented by its two most distinct frames (i.e., non-specular and specular reflection; refer to the Supplementary Material to see the dynamic behavior of the actual video samples).

**Figure 7.**
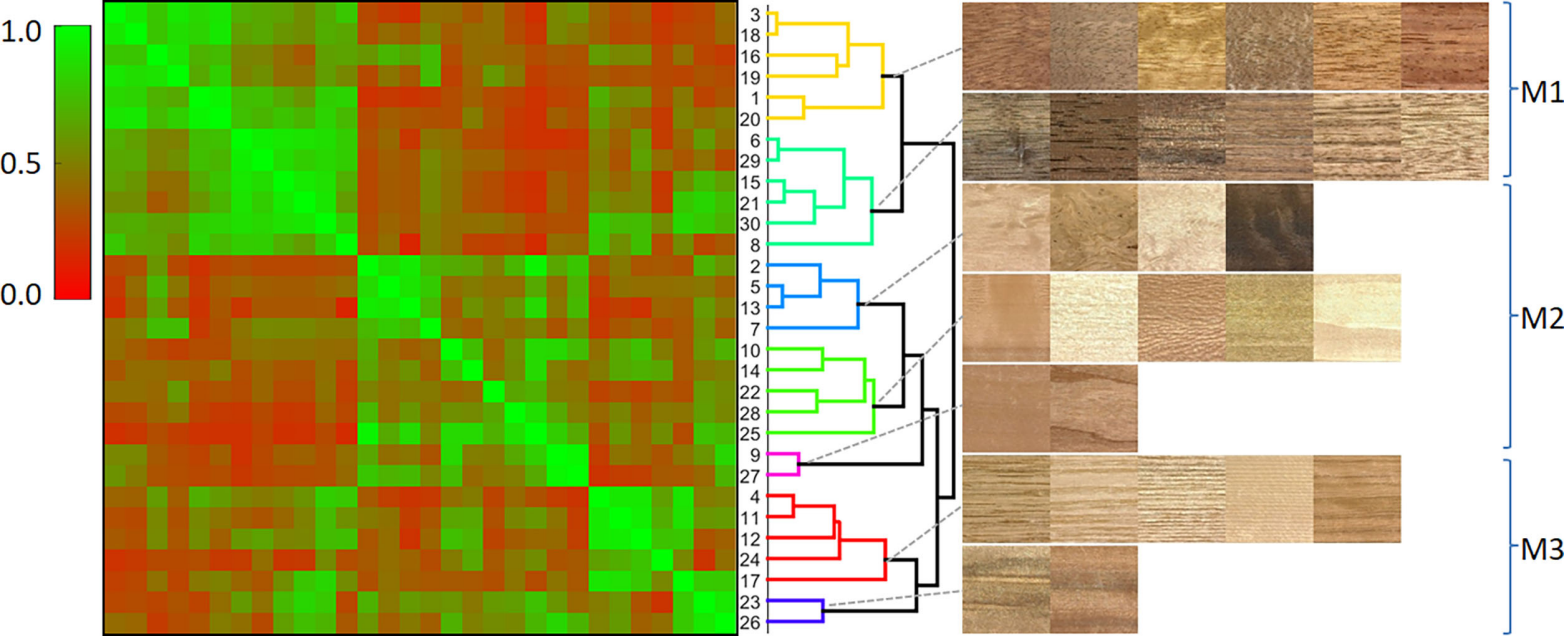
Estimated pairwise similarity matrix with samples ordered based on hierarchical clustering and the depiction of the corresponding samples in the individual clusters.

The full pairwise similarity matrix of the wood samples that we obtained from the estimated embedding (see Hebart et al., 2020) is shown in Figure 7. For similarity matrix computation was used Equation (1) from the Supplementary Material. We used hierarchical clustering (based on weighted average L2-norm) to cluster similar samples together, showing that samples had approximately three main visual modes, which might be visually interpreted as samples having high levels of roughness or contrast (M1), low spatial frequency (M2), and prominent directional elements (M3). These modes are also present in individual similarity dimensions in Figure 6, where M1 is represented by dimensions 1, 5, and 9; M2 by 2 and 6; and M3 by 4, 8, and 3. Note that similar modes were also found using the Louvain community detection method (Blondel, Guillaume, Lambiotte, & Lefebvre, 2008), as reported in Section 2 of the Supplementary Material.

### Discussion

The analysis of participants’ similarity judgements using the VICE model provided us with nine similarity dimensions of wood. However, even though visualizing the embedding by ranking samples within each dimension may provide some intuition about the meaning of the dimensions, it is not clear whether these intuitions are the best description of the respective dimensions. For this reason, we performed a second comparative experiment relying on standard attributes' ratings on a Likert scale.

## Experiment 2

The main goal of the second experiment was to obtain perceptual judgements for all wood samples for a set of visual appearance attributes widely used in the field of material perception. By being able to describe our samples in terms of these specific perceptual attributes, we aimed to provide a more valid interpretation of the similarity dimensions from the first experiment–and a corresponding understanding of the main visual cues that naive observers use to describe and discriminate between types of wood.

### Methods

#### Participants

Forty-five volunteer observers participated in the online experiment (age data were not collected). All participants reported normal or corrected-to-normal vision and no color vision impairments. On average, the experiment took 22.0 minutes (SD = 17.6).

#### Apparatus and stimuli

The stimuli used in Experiment 2 were the same as in Experiment 1.

#### Procedure

Participants were presented with 30 trials, each showing one of the sample videos from Experiment 1. The resolution of each stimuli image was 920 × 600 pixels. To make the task easier for participants, all other materials were simultaneously presented for comparison at a smaller scale at the top of the screen, as shown in Figure 4b. Participants rated each material on ten visual appearance attributes (brightness, glossiness, colorfulness, directionality, complexity, contrast, roughness, patchiness/regularity, line elongation, and spatial scale), using a visual analog scale. The attributes were selected based on a review of previous research (Tamura, Mori, & Yamawaki, 1978; Rao & Lohse, 1996; Fleming et al., 2013; Tanaka & Horiuchi, 2015, Nordvik et al., 2009) and salient differences between samples identified by the experimenters. For the participants, the meaning of each visual attribute was explained with a short sentence (e.g., brightness: “*How bright is the material in comparison with the others?*”). Also, the endpoints of each scale were labeled (e.g., brightness: “dark” and “bright”). A full description of each visual attribute and the corresponding endpoint labels is provided in Section 4 of the Supplementary Material.

All attribute scales were on the screen simultaneously, and at the start of each trial all sliders were set to the center of each scale. Only after moving all sliders, participants could proceed to the next trial.

#### Data analysis

Again, a post-hoc analysis of monitor settings showed a sufficient minimum screen resolution of 980 × 768 pixels. The inter-rater agreement was determined using the intraclass correlation coefficient (ICC; Koo & Li, 2016, with two-way random effects, based on mean rating and consistency). More detailed analysis of participants’ responses is provided in Section 5 of the Supplementary Material. We computed the similarity matrix from observers' ratings using equations (2) and (3) in the Supplementary Material. Note that as attributes’ values are collected on a 100-point scale, the similarity in a particular dimension is expressed by the 100 minus absolute difference of rating values (2), whereas the joint similarity matrix is the root mean square individual-attribute similarity (3).

### Results

**Figure 8.**
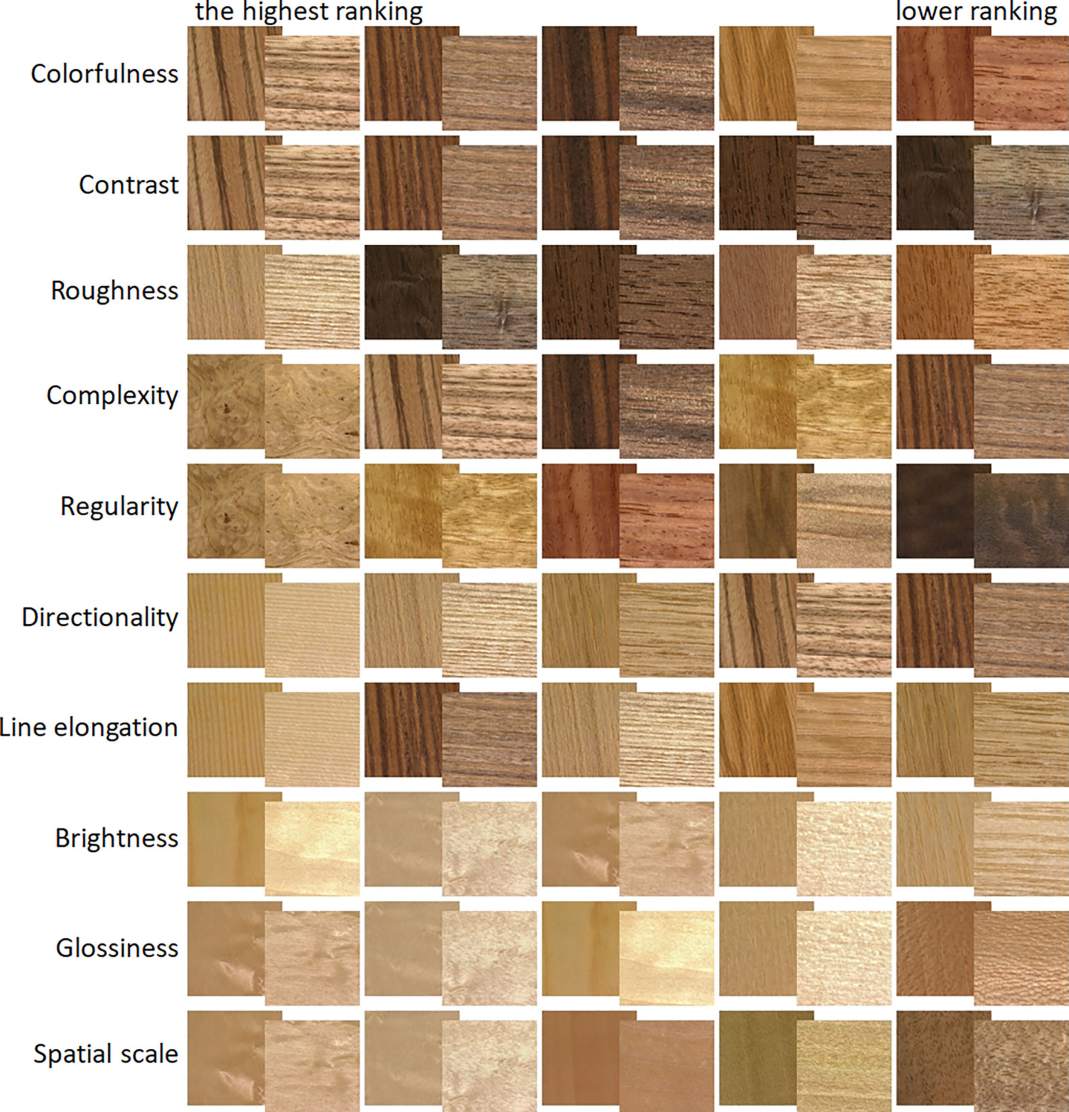
Five samples for each rating dimension rank-ordered based on average rating responses. Each video sample is represented by both the most non-specular and most specular condition. See the right side of Supplementary Video S2.

The rating responses for each attribute formed unimodal distributions with mean values close to the central point (45.8 to 59.5) and similar SD values (21.7 to 29.6). The ICC indicated excellent reliability (ICC > 0.898) for all attributes, but *spatial scale* where ICC = 0.659 indicated only moderate reliability. Samples with the highest rating responses for each rating dimension are shown in Figure 8, with visually intuitive results in the majority of dimensions (again, with the exception of *spatial scale*).

**Figure 9.**
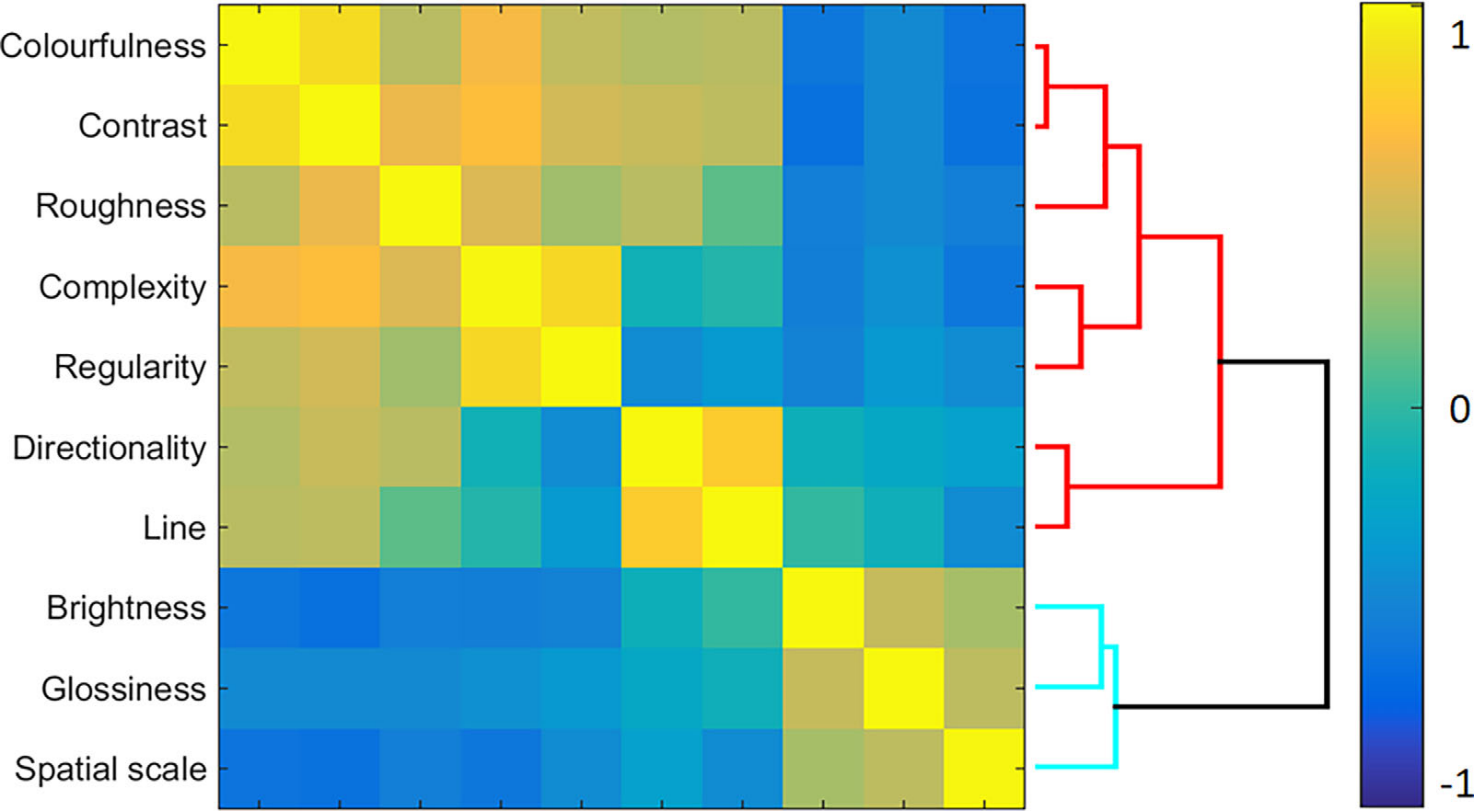
Interclass similarity, computed as Pearson correlation across all samples, with the dendrogram showing the results of hierarchical clustering of attributes.

Note that these examples also suggest similarities between rating dimensions (i.e., overlap in samples for, e.g., *colorfulness* and *contrast*). To measure these interclass similarities, we computed Pearson correlations for mean rating values across all 30 samples. As shown in Figure 9, we observe a high positive correlation between *colorfulness-contrast, directionality-line elongations,* and *complexity-patchiness/regularity*. On the other hand, a high negative correlation is observed for *brightness-colorfulness* and *brightness-contrast*. These similarities are also evident at the level of individual samples, as is shown in Figure 13a, which shows similarity matrices for individual rating dimensions. See Supplementary Video S2 with material samples ranking as a function of dimensions loadings of VICE (left) and rating responses having the highest and the lowest values.

### Discussion

Our rating experiment provided reliable and visually intuitive data on the selected visual appearance attributes, but also highlighted mutual dependencies between some of the attributes. This suggests that our samples can be described by less than 10 attributes, that is, the latent visual dimensionality of our samples is lower than 10. In the next section, we compare an overlap between samples’ embeddings obtained from the similarity and rating experiments.

## Interpretation of similarity dimensions

As the meaning of the similarity dimensions discovered by the VICE model are not known, we used cross correlation and multilinear regressions between appearance ratings and similarity judgements as well as between their respective similarity matrices. This allowed us to assign meaning to the similarity dimensions by relating them to the meaningful appearance ratings.

### Cross-correlation of similarity and rating dimensions

In this section, we compare the reference similarity matrix obtained from the similarity judgments (Figure 7) with the (1) similarity matrices obtained from individual appearance attribute ratings, and (2) their combinations using L2-norm and linear regression (also see Figure 2).

Across all appearance attributes, the mean correlation between similarity matrices from ratings and similarity judgements is relatively low (Pearson *R* = 0.269 exclusion of matrix diagonal), with the highest correlations for *directionality* (*R* = 0.378) and *roughness* (*R* = 0.412). This confirmed our expectation that the similarity embedding cannot be explained using a single rating attribute.

**Figure 10.**
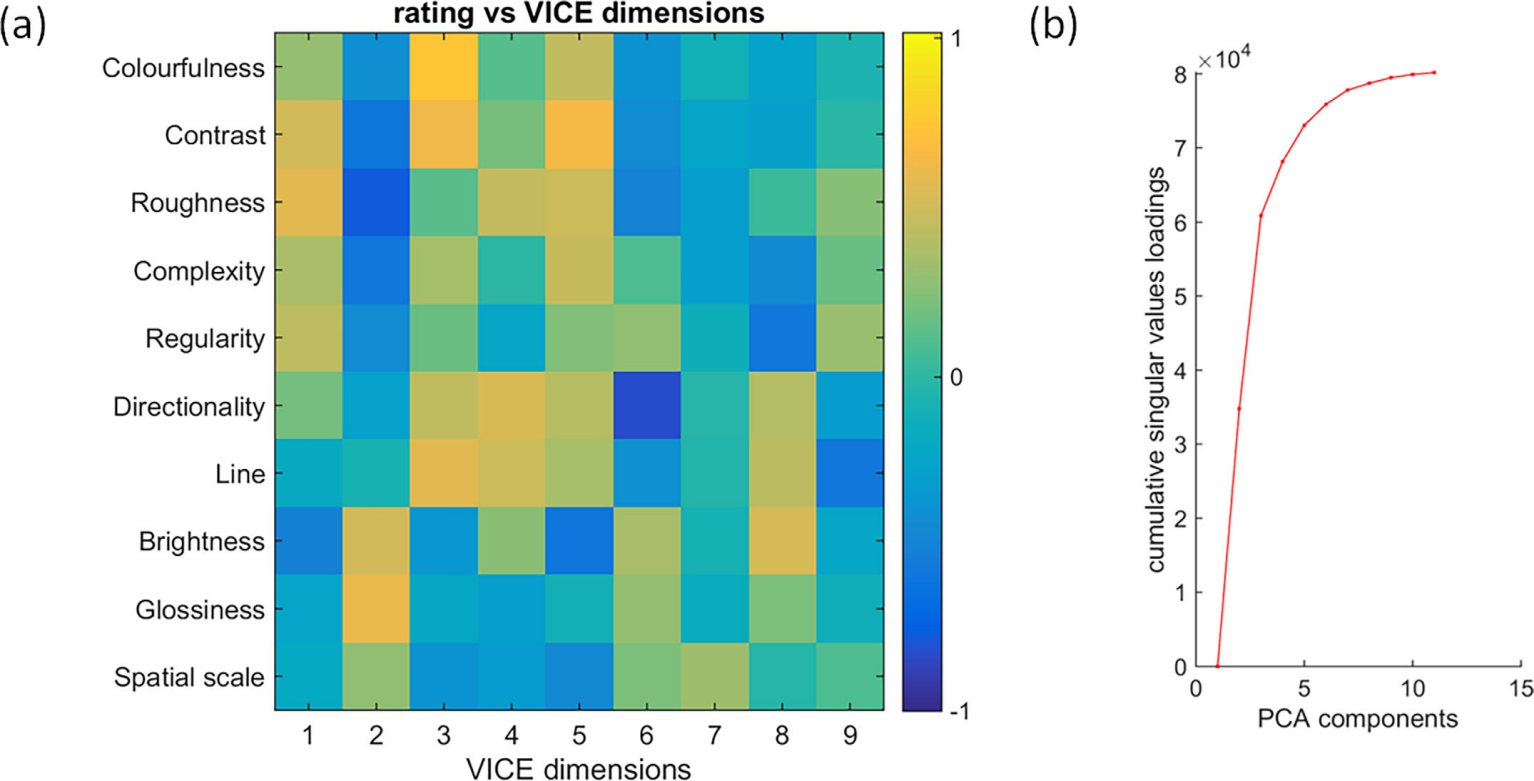
(**a**) Correlations of rating attributes (rows) to similarity dimensions (columns), with negative correlations in red and positive correlations in green (range [−1, 1]). (**b**) Cumulative singular values loadings of PCA computed on correlations across rating attributes (**a**), an indication of the effective perceptual dimensionality of the dataset.

For a direct correlation between all attributes’ ratings and all VICE similarity dimensions, see Figure 10a. The highest positive correlation was *R* = 0.739 and the highest negative correlation was *R* = −0.812. Notably, similarity dimensions 1, 3, 4, and 5 show similar patterns of correlation to rating attributes *colorfulness*, *directionality*, *complexity,* and *roughness*. On the other hand, similarity dimension 7 is not correlated strongly with any rating attribute, which suggests that none of them can explain the visual appearance captured by this particular dimension. To test whether the similar pattern of correlations across similarity dimensions follows from a strong dependency between individual rating attributes, we computed principal component analysis (PCA) on our rating data. Figure 10b shows that only five PCA components explain 91.1% of the variance, suggesting that the effective number of main perceptual dimensions for our set of wood samples is above 5. We confirm this hypothesis by using a statistical approach to estimate the number of data dimensions based on triplet embedding accuracy of ordinal triplets embedding (Künstle, von Luxburg, & Wichmann, 2022)—which identifies six as the inherent dimensionality of our data (see details on this analysis in Section 3 of the Supplementary Material). This is also supported by the steep drop of similarity embedding factor loadings after the fifth perceptual dimension (Figure 5b). However, note that VICE is not optimized to obtain a low number of perceptual dimensions but to retrieve sparse and non-negative dimensions. Specifically, the purpose of the VICE algorithm is to find an overarching set of dimensions used across all comparisons, even if for any given comparison, only a subset of these came into play. For example, for one triplet of materials, color might be especially important, but not for another. Our analysis of the five best VICE models revealed that this dimensionality is quite stable and at least five (9, 8, 8, 9, 5). The effect of removing individual dimensions on model accuracy, as well as an analysis of model stability, is provided in the Supplementary Material.

**Figure 11.**
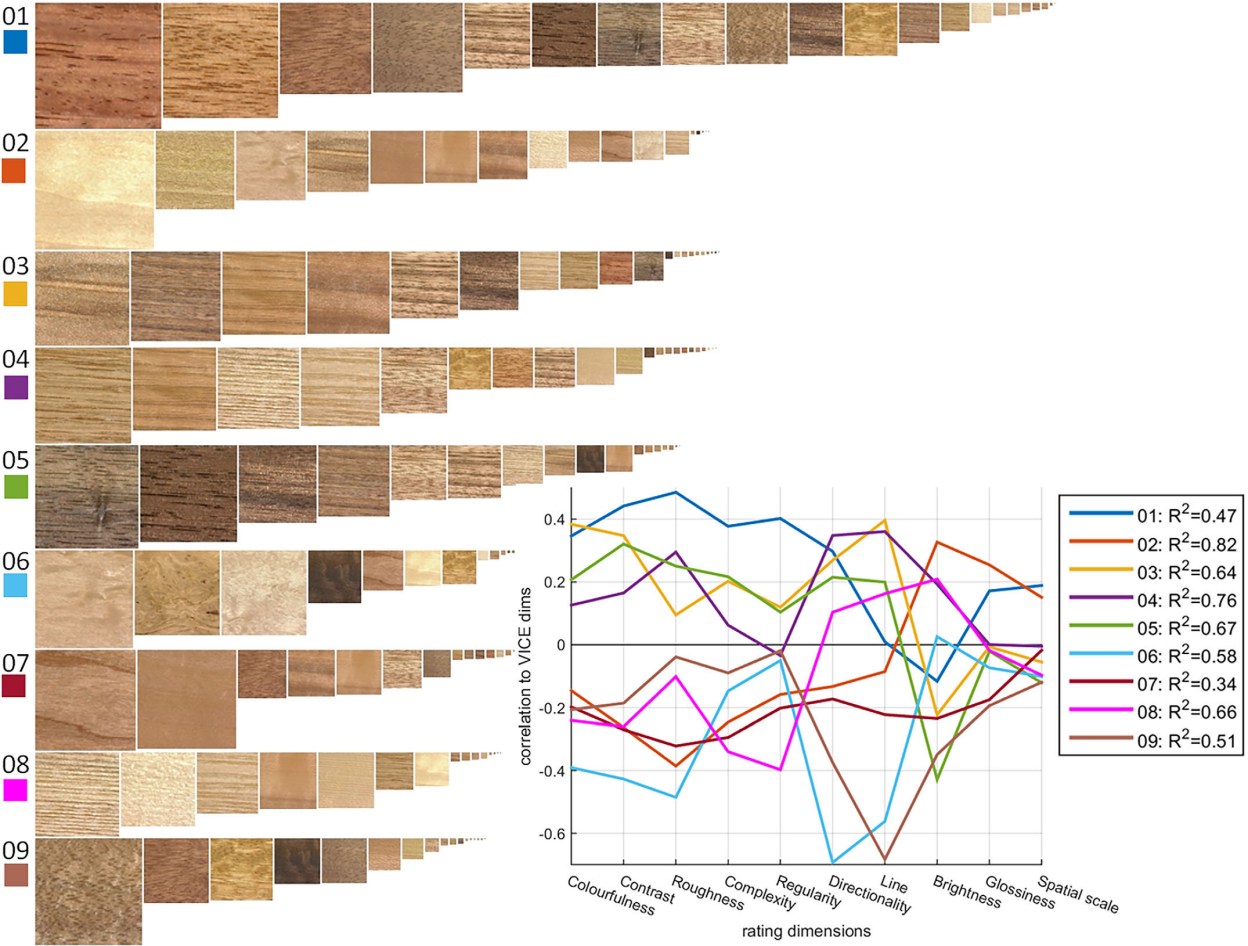
Sample rank-ordered by embedding values in VICE similarity dimensions. Inset: Correlations between similarity (VICE) and rating attributes, obtained from linear regressions (*R*^2^ scores provide information on how well the linear regression using rating attributes explained individual similarity dimensions. See Supplementary Video S3.

A more quantitative comparison between similarity dimensions and rating attributes is shown in Figure 11. For each similarity dimension, we ordered and scaled samples according to their embedding values along each dimension. The inset shows how well the variation in each similarity dimension is correlated with different rating attributes. Here, we observe similar patterns for dimensions 1, 3, 4, and 5, while dimension 7 is virtually constant across rating attributes. *R*^2^ scores in the legend demonstrate how well each similarity dimension can be predicted by linear regression of rating attributes.

**Figure 12.**
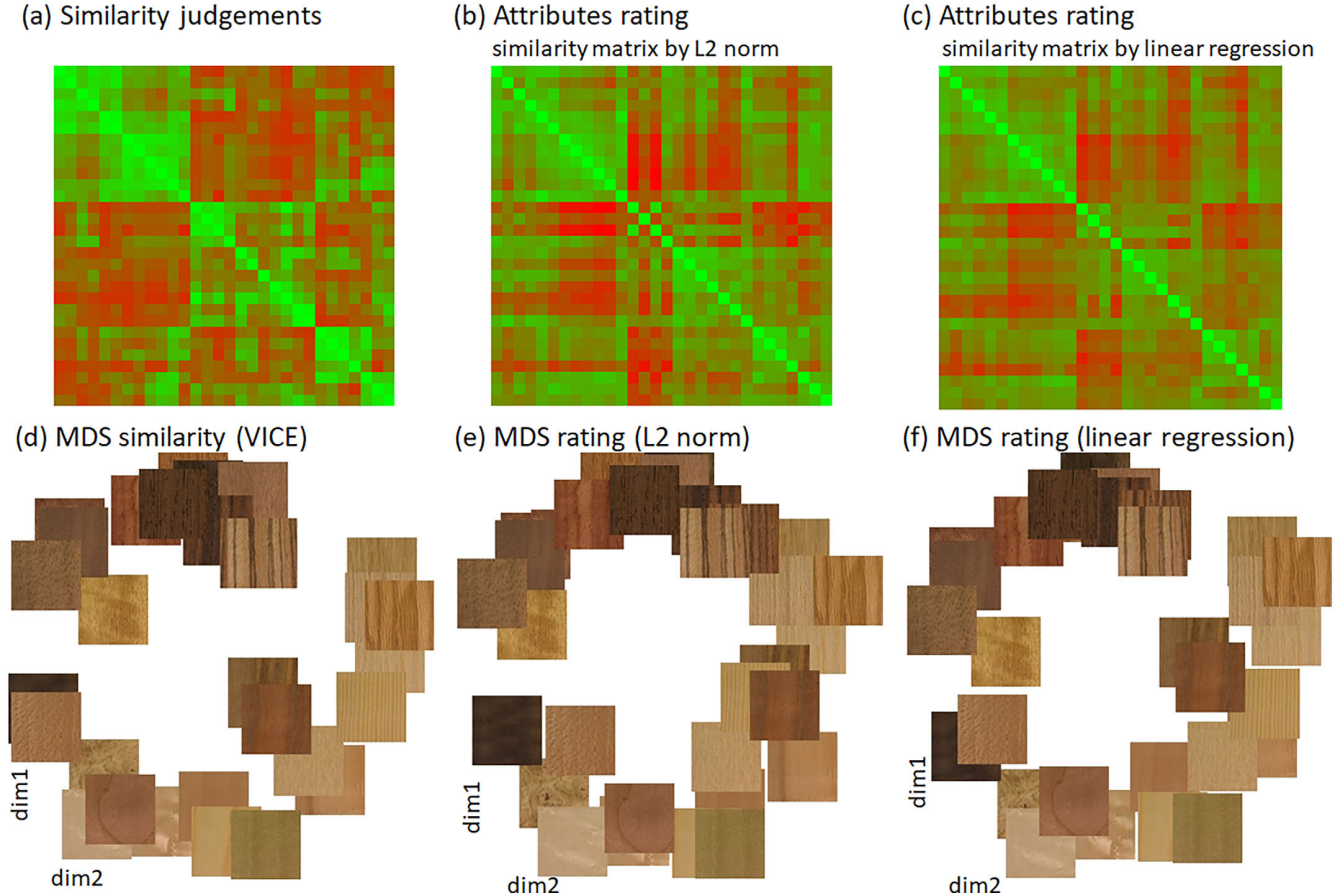
A comparison of similarity matrices obtained by (**a**) similarity judgements and (**b**, **c**) ratings using L2-norm and linear regression, respectively. The correlation between matrices is for (**b**) *R* = 0.627 (*R*^2^ = 0.393) and for (**c**) *R* = 0.720 (*R*^2^ = 0.519). Corresponding embeddings of samples in the first two MDS dimensions for (**d**) similarity judgement and (e, f) ratings (after Procrustes alignment). The Supplementary Material also features the exact mapping of wood sample locations in the MDS space.

To compare the results obtained from the two experiments, we computed the rating similarity matrix as L2-norm across all attributes (see Equations 2 and 3 in the Supplementary Materials). This shows us how well combined ratings can predict the VICE similarity matrix without any a priori information or weighting (Pearson correlation instead of L2-norm yields very similar results). A direct correlation between similarity matrices obtained from similarity judgement and attributes rating using L2-norm (excluding diagonal elements) was *R* = 0.628 (*R*^2^ = 0.394). The matrices are shown in the first row of Figures 12a and 12b.

We used multidimensional scaling (MDS) (Carroll & Arabie, 1998) on the VICE similarity matrix to visualize the embedding of wood samples in three-dimensional space. The MDS projection of samples onto the first two dimensions is shown in Figure 12d. In line with our visual interpretation of the three main visual modes in Figure 7, the first MDS dimension can be interpreted as related to roughness, the second to directionality and the third to spatial frequency. For clarity, we also included these plots with the video samples as presented to observers. We compared MDS results over the similarity matrices and coordinates of all 30 samples for the first two MDS dimensions after Procrustes alignment, which are shown in Figure 12e. For MDS of VICE similarity matrix into all three dimensions, see the top part of the Supplementary Video S4.

### Prediction of similarity matrix from rating attributes

**Figure 13.**
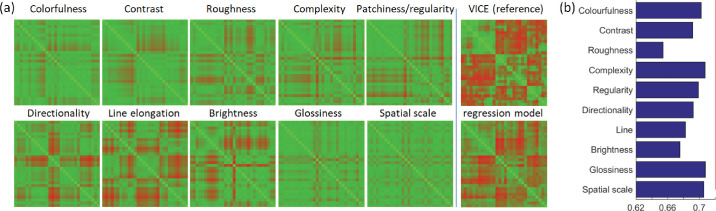
(**a**) Similarity matrices of individual rating attributes compared to the VICE similarity matrix and the result of the linear combination of the 10 rating similarity matrices. (**b**) Results of leave-one-out regression analyses showing the respective drops in correlation below the red baseline due to individual attributes removal.

Beyond simple correlations between individual ratings and similarity dimensions, we can test how well a combination of rating attributes predicts similarity judgements. To this end, we used multilinear regression to predict the similarity judgement matrix in Figure 12a by a linear combination of the rating attribute similarity matrices shown in Figure 13a. The matrices’ diagonals were kept to anchor scaling. The regression model explains about 52% of the variance in similarity judgements (*R* = 0.720, *R*^2^ = 0.519) while still preserving the major similarity modes, as shown in Figure 13a. To evaluate the importance of individual rating attributes for the reconstruction, we performed leave-one-out regressions and the resulting drops in explained variance. Figure 13b shows that the most important attributes are roughness, brightness, and line. A comparison of the obtained MDS over the similarity matrices and coordinates of all 30 samples for the first two MDS dimensions after Procrustes alignment is shown in Figure 12f. Also, see Section 6 of the Supplementary Material for samples alignment according to MDS and Supplementary Video S4, comparing three MDS dimensions of similarity judgements similarity matrix (top) with its linear regression using rating attributes (bottom).

### Mapping between similarity dimensions and rating attributes

**Figure 14.**
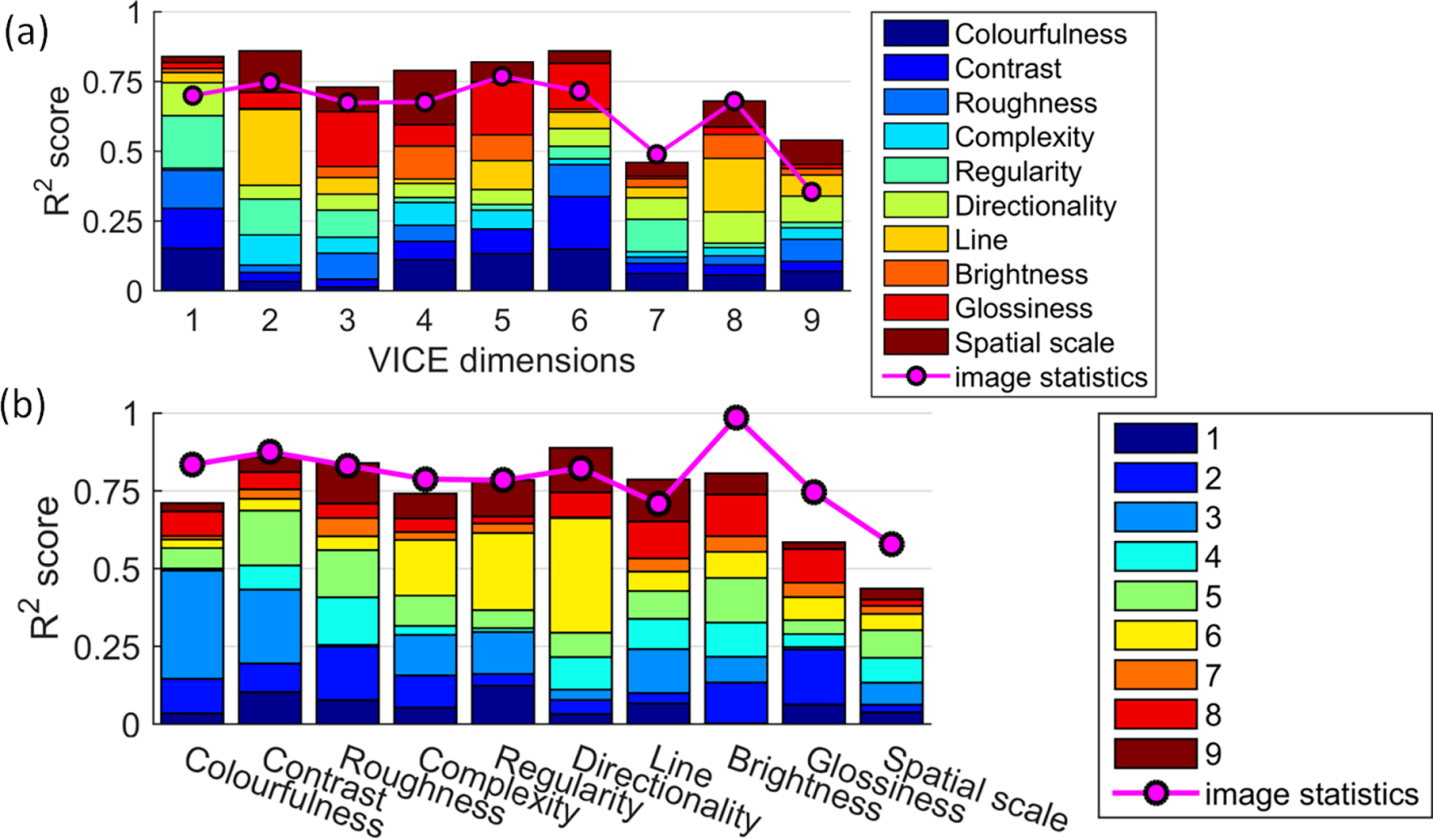
*R* ^2^ scores of regression of similarity dimensions by rating attributes (**a**), and regression of rating attributes by similarity dimensions (**b**) are shown as the total height of stacked bars. Individual colors in the bars represent the contribution of regression values of individual rating attributes (**a**)/VICE dimensions (**b**). Overlaid magenta graphs show *R*^2^ scores of VICE dimensions (**a**)/rating attributes (**b**) when represented by regression of computational image statistics.

Because there is no straightforward one-to-one mapping between VICE similarity dimensions and subjective rating attributes, in this section, we analyzed how well each similarity dimension can be represented by a combination of multiple rating attributes and vice versa. First, we used linear regression to predict individual VICE similarity dimensions by a linear combination of the rating attributes. The linear combination of ratings can well explain individual similarity dimensions, with an average of *R* = 0.851 (R^2^ = 0.731). In Figure 14a, *R*^2^ scores of similarity dimensions represented by the regression model are shown as total bar height. All dimensions (with the possible exception of 7 and 9) can be well explained by a combination of rating attributes (*R*^2^ scores > 0.7).

As reported previously, dimension 7 is not well predicted by any of the rating attributes. This might be for two reasons: either none of our predefined attributes captures the same visual appearance as that similarity dimension, or there is a general bias in our rating data that is introduced by a particular interpretation of the to-be-rated attributes. For instance, *line elongation*, *patchiness/regularity,* or *spatial scale* might have different meanings at different frequency scales. For instance, samples 22 and 30 (see Figure 3) both share a fine detail structure and a distinct low-frequency stripy pattern. As a result, observers might be confused as to whether these attributes should be evaluated on a fine or coarse scale, resulting in overall ambiguous ratings.

In the stacked bars of Figure 14a, we plot linear regression values in different colors to visualize the contribution of rating attributes to each of the similarity dimensions. For example, dimension 1 is strongly negatively related to colorfulness and roughness but strongly positively related to contrast, regularity, and directionality, whereas dimension 5 represents materials that were judged low on line elongation, brightness, and glossiness (see samples rank ordering along dimensions in Figure 11).

Similarly to this analysis, we applied linear regression to predict subjective rating attributes by a linear combination of the VICE similarity dimensions. The mean correlation between subjective ratings and their linear model prediction is *R* = 0.859 (*R*^2^ = 0.737). Results for individual rating attributes are reported as total bar height in Figure 14b, demonstrating very good performance of VICE dimensions in reproduction of all ratings (with the possible exception of *glossiness* and *spatial scale*). When we reduced the number of VICE dimensions used for regression to only the two or three most important, based on absolute values of their regression coefficients, we still obtained reasonable mean prediction performance across all attributes *R* = 0.718 (*R*^2^ = 0.515). The regression coefficient values for individual VICE dimensions are shown in different colors within stacked bars in Figure 14b. For instance, attribute *colorfulness* is mainly represented by VICE dimension 3, whereas *regularity* and *directionality* are strongly represented by VICE dimension 6.

We have determined that the VICE similarity dimensions provide valuable information, effectively predicting the majority of rating attributes. However, we discovered a surprising lack of significant overlap between the VICE dimensions and our rating attributes, leading to a non-intuitive interpretation of these dimensions. We hypothesize that the use of dynamic stimuli in our experiment caused individual dimensions to encompass multiple visual cues used by observers, making their complete disentanglement an open challenge. The diminished performance of VICE dimensions in predicting attributes such as glossiness and spatial scale might be attributed to their low intuitiveness for observers during the rating study. Alternatively, it could be due to their overall low importance, as indicated by the limited descriptive performance of the VICE dimensions.

### Relationship between perceptual dimensions and image statistics

To relate similarity dimensions to computational statistics, we used a number of standard image statistics used in texture synthesis related to human low-level perception of textures (Portilla & Simoncelli, 2000, Motoyoshi et, 2007), namely *minimum, maximum, mean, variance, skewness,* and *kurtosis*. We supplied additional statistics evaluating image directionality (Maskey & Newman, 2021) and frequency content in three bands (*low, mid*, and *high* frequencies) computed from PSD of the image converted to the Fourier domain. The final values of statistics were averaged across all frames of the movie sequence. We used these statistics for linear regression of VICE similarity dimensions and attributes ratings, and *R*^2^ scores of results are shown as magenta overlaid plots in Figures 14a and 14b, respectively. We observe similar values of *R*^2^ scores to those obtained from ratings regression, and in general all similarity dimensions, except 7 and 9, can be represented reasonably well using our statistics. The mean *R*^2^ score across VICE similarity dimensions was 0.65 (*R* = 0.73), and across rating attributes 0.79 (*R* = 0.89).

Detailed plots of all regression coefficients are shown in Section 8 of the Supplementary Material. Also, see Supplementary Video S5, comparing three MDS dimensions of similarity judgements similarity matrix (top) with a similarity matrix obtained as L2-norm of all 11 computational statistics (bottom). The video demonstrates reasonable prediction performance of the statistics.

## General discussion

In this study, we set out to identify core perceptual characteristics of wood. Characterizing the visual appearance of wood is complex because of the variety of factors like color, grain patterns, fine-scale relief, and reflectance behavior. Accordingly, a description in physical terms requires very high-dimensional measurements that capture the image projected by the material surface across many lighting conditions and viewing angles. Yet, we reasoned that when human observers are asked to compare samples—or judge the appearance of a single sample—they would rely on a relatively small number of perceptual dimensions that together summarize the overall “look” of each surface and its texture—what we might call a “visual signature” of the material (Sharan et al., 2013; Schmidt et al., 2022).

Here, we wanted to estimate such an internal multidimensional representation by asking observers to make comparisons between samples. A secondary goal was to test the extent to which different methods of probing this putative representation yielded similar embeddings of the material samples. We reasoned that if observers draw on shared, core perceptual dimensions to judge the appearance of wood, it should be possible to probe this representation using distinct tasks.

To test this, we performed two experiments using movies of thirty samples of different wood veneers, rotating in such a way as to reveal both non-specular and specular appearance modes. In the first experiment, we took a data-driven approach, asking participants to make relative similarity judgments in a 2AFC task, from which we sought to derive underlying perceptual dimensions using the VICE algorithm (Muttenthaler et al., 2022). In the second experiment, we defined a set of 10 appearance characteristics and asked participants to rate each sample in terms of all 10 characteristics, effectively directly stating the location of each sample in a 10-dimensional appearance space. Our main findings can be summarized as follows:

• In Experiment 1, the VICE algorithm revealed that nine similarity dimensions could account for 75% of the variance in the similarity judgments, consistent with the notion of a low-dimensional “visual fingerprint” summary representation of their appearance.
• In Experiment 2, participants were consistent in their judgments of the 10 appearance characteristics, suggesting agreement about the embedding of samples relative to one another.
• Despite substantial differences in the tasks between the two experiments, we nevertheless found a degree of overlap between embeddings of the samples derived from the two tasks (over 50% shared variance), providing further evidence for a core representation of wood materials, with similar-looking samples close to one another, and more distinct ones further away from one another within the multidimensional appearance space.
• The consistency between the two experiments can also be demonstrated by approximating the dimensions inferred from Experiment 1 as a weighted linear combination of the predefined attributes ratings in Experiment 2.
• Finally, a set of quite simple low-level image features, designed to capture similar appearance characteristics as the rating attributes predict the ratings and similarity dimensions surprisingly well, using simple linear regression. Although these image features will not be the exact quantities that the visual system uses to represent and compare the wood samples, this shows how we can use straightforward image-computable models to predict perceived differences in appearance (under constant viewing conditions). This has potential practical applications in many areas.

Our study also provides a proof-of-principle demonstration that it is possible to establish embeddings of items from a single basic-level category (here: wood) within a perceptual space using either a subset of all possible similarity comparisons, or through direct rating of particular features. The study differed from previous investigations in the use of movies rather than static images, capturing a wide range of appearances for each sample, and in the comparison between similarity and appearance ratings.

### Limitations and future directions

Although our study provides a first proof-of-principle for identifying perceptual dimensions within a single material category, there are a number of important limitations of the approach, which we consider here.

#### Limited number of wooden samples

The stimulus set considered here consisted of only thirty samples of different wood veneers, as listed in Table 1. This is one of the largest sets of wooden samples used in a psychophysical analysis to date, and we carefully selected this set from a catalogue of 200 wood veneers to provide as broad and uniform a range of appearances as possible. However, including a larger number of samples would necessarily provide additional information about the embedding and would potentially reveal additional perceptual dimensions by covering a wider range of appearances. On the other hand, including more samples would have made our similarity experiment much more demanding. Thus, to validate our sample selection, we repeated the attributes rating experiment on a different set of thirty samples and obtained a much lower variability in image statistics and rating responses compared to the main dataset. This suggests that we were successful in picking samples with a high variability. It would also be particularly interesting to include multiple samples of each species (see Table 1) in future work to capture within-item variability as well. We would expect that although different samples would be clearly discriminable, generally, they would tend to occupy very close locations within the multidimensional perceptual space.

#### Limited observation and illumination geometry

By using dynamic stimuli, in contrast to previous studies, which tended to offer only a single view of each sample, we were able to provide observers with some information about how the appearance of the samples changed depending on viewing conditions, including both specular and non-specular conditions. Nevertheless, this still represented a limited subset of all possible lighting-sample-viewer configurations. We had to limit camera and light trajectories so that movies were of reasonable duration. Based on pilot work with a range of different sampling parameters, we identified a rotation that was of acceptable durations, and that was intuitive for observers. As the appearance of wood does not typically change much with polar angle, we limited polar viewing angles to 45° and changed azimuthal angles only. A comparison of image histograms from our videos with those of the full BTF for the same material (at polar angles 45°, including over 400 images for different combinations of illumination and view azimuthal angles) provided mean differences of χ^2^ lower than 0.10. This leaves us confident that the selected views were representative of the overall appearance.

#### Dynamic range artefact in some stimuli

For three of 30 stimuli there was a briefly visible flickering artefact for a few (three or four) frames around the specular angle. This resulted from an error in the tone mapping process of high dynamic range data, but because it was limited to only a few samples and the effect was small, we expect it to have a limited impact on the results reported here.

#### Limited size of samples

On a related point, the visible area of the samples was around 50 × 50 mm. This size was selected to deliver fine surface details. On the other hand, for certain species, there may be low-frequency content that was excluded by the small size. To compensate for this during video acquisition, the location of the captured area on the veneer specimen was carefully selected to demonstrate the main sample's characteristics. A similar comparison of histogram statistics with BTF data over a large-scale of image plane resulted in similarly low differences in histograms, again indicating that the patch was representative of the sample as a whole. Of course, our dataset cannot describe visual behavior going beyond our sample size (i.e., too low spatial frequencies in texture or slow gradient change over the sample).

#### Limited coverage of triplets for similarity judgements

In Experiment 1, we measured only a small subset of all possible stimulus triplets. Specifically, our experiment had a coverage of 10%, which is nevertheless far greater than the less than 2% coverage used in other studies using related data analyses (Hebart et al., 2020). On the other hand, our number of samples is considerably lower, greatly reducing the number of necessary trials. We followed the recommendations in (Haghiri, Wichmann, & von Luxburg, 2020) to estimate the number of judgements, although future studies could potentially increase the coverage further for small stimulus sets like ours.

#### Stability of similarity dimensions

Statistical inference methods like VICE are stochastic, so repeated runs of the algorithm on the same data can deliver slightly different outcomes. This naturally raises questions about the stability and interpretation of the outcome. We tested a wide range of hyperparameter values, and found the values we used delivered representative results. Importantly, although the exact number of VICE similarity dimensions varied across runs, the meanings of those dimensions (i.e., the loadings across samples) were highly conserved. This, along with the high extent to which the dimensions could predict similarity ratings, gives high confidence that the analysis delivered robust results. Increasing the number and diversity of samples, as well as the coverage, would lead to even greater stability, although with obvious practical costs. It is nevertheless important to emphasize that in interpreting results on small and constrained stimulus sets like our, greater emphasis should be placed on the *embedding of items* within the multidimensional space than on the precise number or direction of the dimensions returned by VICE (or related algorithms). The convergence between the ratings and the VICE analysis supports this view.

#### Intuitive interpretability of individual similarity dimensions

Although some studies (e.g., Hebart et al., 2020; Josephs et al., 2023; Schmidt et al., 2022) have found that analyses similar to VICE deliver dimensions that are highly intuitively interpretable, in our case, most of the dimensions appeared to be better understood as weighted combinations of several intuitive factors. This can be seen in Figure 11, for example, in which samples are ranked by their values of the nine similarity dimensions returned by VICE. Some of the dimensions seem to capture intuitive concepts. For example, dimension 4 appears related to stripiness, and this is consistent with the high loading of the “Directionality” and “Line” features in the multiple regression for this feature. Dimension 6, in contrast, seems to be approximately the opposite, with an emphasis on samples with turbulent texture patterns rather than linear grain. However, for most of the other dimensions, the interpretation is less intuitive. This is likely due to the small and constrained sample set. With diverse image sets that span the entire range of commonly occurring objects, for example (Hebart et al., 2020), almost all samples will have near-zero values of any given attribute, although there are still sufficient numbers of images with high values to enable a dimension to emerge from the analysis. Indeed, such datasets are particularly well suited for seemingly meaningful individual dimensions to be recovered by the sparse nonnegative matrix factorization. By contrast, within-category samples, as in our experiments, tend to involve characteristics that are more uniformly distributed across samples. This is likely to be one of the reasons that the recovered similarity dimensions were composites of multiple factors. Nevertheless, again it should be noted that we place greater emphasis on the embedding of items within the space than on the exact orientation of the underlying dimensions.

#### Choice of rating dimensions

There are practical limits to the number of appearance attributes that participants can feasibly be asked to rate for each sample. As with the majority of previous perceptual studies of wood surfaces (Nakamura et al., 1999; Nordvik et al., 2009; Fujisaki et al., 2015; Manuel et al., 2015, Wan et al., 2021), we preselected a list of visual properties in our rating experiment. This list, of course, is likely to be incomplete as there are potentially infinitely many ways of describing samples, including those that may make intuitive visual sense but which cannot easily be put into words. Nevertheless, we find that this set of similarity dimensions leads to intuitive and repeatable judgments, which are sufficient to capture an embedding of the samples similar to that revealed by the similarity ratings and VICE analysis. Future studies could also ask participants, rather than the experimenters, to provide terms that describe important appearance differences between samples, which other participants would then rate (see, e.g., Van Assen, Barla, & Fleming, 2018).

## Conclusions

Our study sought to identify core perceptual dimensions underlying the appearance of wood. Using 30 movies of rotating planar wooden veneer samples, we asked participants to judge the similarity between items and rate each sample along 10 predefined attributes. Our main findings can be summarized as follows:

1. We find that woods have complex appearances. To account for similarity judgments between samples in our dataset, between five to 10 distinct perceptual dimensions need to be considered.
2. The space of wood appearances depends on the method used to probe it. Specifically, the material embeddings resulting from the similarity judgments and attribute ratings were substantially correlated but not identical.
3. Individual similarity dimensions could be expressed as a weighted linear combination of the following 10 attributes: brightness, glossiness, colorfulness, directionality, complexity, contrast, roughness, patchiness/regularity, line elongation, and spatial scale.
4. Certain attributes appeared to be particularly important to participants when making similarity judgements, especially those related to the roughness, directionality, and scale of visual features.

Together these findings suggest a core internal representation of the samples, capturing the overall “look” of the samples in a relatively small number of similarity dimensions. The results not only reveal the core dimensions underlying the perception of wood, they also provide a proof of concept demonstration for how perceptual dimensions underlying judgments within a single basic-level category can be probed using multiple tasks.

## Supplementary Material

Supplement 1

Supplement 2

Supplement 3

Supplement 4

Supplement 5

Supplement 6

Supplement 7

## Supplementary material

Supplementary Video S1. 30 test wood video sequences used in the experiments.

Supplementary Video S2. Rank ordered samples (left) according to loadings values of similarity dimensions, (right) mean rating attributes (the five closes and 5 the most distant).

Supplementary Video S3. Rank ordered samples scaled according to loadings values of similarity dimensions.

Supplementary Video S4. Distribution of samples along three MDS dimensions (top) for similarity judgements, (bottom) for rating study.

Supplementary Video S5. Distribution of samples along three MDS dimensions (top) for similarity judgements, (bottom) for computational statistics obtained from image sequence.

Supplementary Video S6. Result of community detection using Louvain method (computed from similarity matrices), distributing samples to three clusters for (top) similarity judgements and (bottom) rating study.

## References

[bib1] Anderson, B. L. (2011). Visual perception of materials and surfaces*.* *Current Biology**,* 21(24), R978–R983. 22192826 10.1016/j.cub.2011.11.022

[bib2] Bell, S., Upchurch, P., Snavely, N., & Bala, K. (2015). Material recognition in the wild with the materials in context database*.* In: *Proceedings of the IEEE conference on computer vision and pattern recognition,* (pp. 3479–3487).

[bib3] Blondel, V. D., Guillaume, J. L., Lambiotte, R., & Lefebvre, E. (2008). Fast unfolding of communities in large networks. *Journal of Statistical Mechanics: Theory and Experiment,* 2008(10), P10008.

[bib4] Bracci, S., & Op de Beeck, H. P. (2023). Understanding human object vision: a picture is worth a thousand representations*.* *Annual Review of Psychology**,* 74, pp. 113–135. 10.1146/annurev-psych-032720-041031 36378917

[bib5] Carroll, J. D., & Arabie, P. (1998). Multidimensional scaling. *Measurement, Judgment and Decision Making,* 179–250.

[bib6] Dana, K.J., van Ginneken, B., Nayar, S.K., & Koenderink, J.J. (1999). Reflectance and texture of real-world surfaces*,* *ACM Transactions on Graphics,* 18(18), 1–34

[bib7] De Leeuw, J. R. (2015). jsPsych: A JavaScript library for creating behavioral experiments in a Web browser. *Behavior Research Methods,* 47, 1–12. 24683129 10.3758/s13428-014-0458-y

[bib8] Ferwerda, J. A., Pellacini, F., & Greenberg, D. P. (2001). Psychophysically based model of surface gloss perception*.* In *SPIE Human vision and electronic imaging vi**,* Vol. 4299, pp. 291–301.

[bib9] Filip, J., Vavra, R., Haindl, M., Zid, P., Krupicka, M., & Havran, V. (2013). BRDF slices: Accurate adaptive anisotropic appearance acquisition. In *Proceedings of the IEEE Conference on Computer Vision and Pattern Recognition* (pp. 1468–1473).

[bib10] Fleming, R. W., Dror, R. O., & Adelson, E. H. (2003). Real-world illumination and the perception of surface reflectance properties. *Journal of Vision**,* 3(5), 3–3. 10.1167/3.5.3 12875632

[bib11] Fleming, R. W., & Bülthoff, H. H. (2005). Low-level image cues in the perception of translucent materials. *ACM Transactions on Applied Perception**,* 2(3), 346–382.

[bib12] Fleming, R. W., Jäkel, F., & Maloney, L. T. (2011). Visual perception of thick transparent materials. *Psychological Science**,* 22(6), 812–820. 21597102 10.1177/0956797611408734

[bib13] Fleming, R. W., Wiebel, C., & Gegenfurtner, K. (2013). Perceptual qualities and material classes. *Journal of Vision**,* 13(8), 9–9. 10.1167/13.8.9 23847302

[bib14] Fleming, R. W. (2017). Material perception. *Annual Review of Vision Science,* 3, 365–388. 10.1146/annurev-vision-102016-061429 28697677

[bib15] Fujisaki, W., Tokita, M., & Kariya, K. (2015). Perception of the material properties of wood based on vision, audition, and touch. *Vision Research,* 109, 185–200. 25576379 10.1016/j.visres.2014.11.020

[bib16] Haghiri, S., Rubisch, P., Geirhos, R., Wichmann, F., & von Luxburg, U. (2019). Comparison-based framework for psychophysics: Lab versus crowdsourcing. *arXiv preprint arXiv:1905.07234*.

[bib17] Haghiri, S., Wichmann, F. A., & von Luxburg, U. (2020). Estimation of perceptual scales using ordinal embedding. *Journal of Vision,* 20(9), 14–14. 10.1167/jov.20.9.14 PMC7533746 32955551

[bib18] Haindl, M., & Filip J. (2013). Visual texture: accurate material appearance measurement, representation and modeling. *Advances in Computer Vision and Pattern Recognition*. London: Springer-Verlag.

[bib19] Hebart, M. N., Zheng, C. Y., Pereira, F., & Baker, C. I. (2020). Revealing the multidimensional mental representations of natural objects underlying human similarity judgements. *Nature Human Behaviour,* 4(11), 1173–1185. 10.1038/s41562-020-00951-3 PMC7666026 33046861

[bib20] Josephs, E. L., Hebart, M. N., & Konkle, T. (2023). Dimensions underlying human understanding of the reachable world. *Cognition,* 234, 105368. 36641868 10.1016/j.cognition.2023.105368 PMC11562958

[bib21] Koo, T. K., & Li, M. Y. (2016). A guideline of selecting and reporting intraclass correlation coefficients for reliability research. *Journal of Chiropractic Medicine,* 15(2), 155–163. 27330520 10.1016/j.jcm.2016.02.012 PMC4913118

[bib22] Künstle, D. E., von Luxburg, U., & Wichmann, F. A. (2022). Estimating the perceived dimension of psychophysical stimuli using triplet accuracy and hypothesis testing. *Journal of Vision,* 22(13), 5–5. 10.1167/jov.22.13.5 PMC9730733 36469015

[bib23] Lewin, M., & Goldstein, I.S. (1991). *Wood Structure and Composition, International Fiber Science and Technology*. Boca Raton, FL: CRC Press.

[bib24] Manuel, A., Leonhart, R., Broman, O., & Becker, G. (2015). Consumers’ perceptions and preference profiles for wood surfaces tested with pairwise comparison in Germany. *Annals of Forest Science,* 72(6), 741–751.

[bib25] Marlow, P. J., Kim, J., & Anderson, B. L. (2012). The perception and misperception of specular surface reflectance*.* *Current Biology**,* 22(20), 1909–1913. 22959347 10.1016/j.cub.2012.08.009

[bib26] Maskey, M., & Newman, T. S. (2021). On measuring and employing texture directionality for image classification. *Pattern Analysis and Applications,* 24(4), 1649–1665.

[bib27] McCamy, C. S. (1996). Observation and measurement of the appearance of metallic materials. Part I. Macro appearance. *Color Research & Application,* 21(4), 292–304.

[bib28] Motoyoshi, I., Nishida, S. Y., Sharan, L., & Adelson, E. H. (2007). Image statistics and the perception of surface qualities. *Nature**,* 447(7141), 206–209. 17443193 10.1038/nature05724

[bib29] Muttenthaler, L., Zheng, C. Y., McClure, P., Vandermeulen, R. A., Hebart, M. N., & Pereira, F. (2022). VICE: Variational Interpretable Concept Embeddings. *Advances in Neural Information Processing Systems,* 35, 33661–33675.

[bib30] Nakamura, M., Masuda, M., & Shinohara, K. (1999). Multiresolutional image analysis of wood and other materials. *Journal of Wood Science,* 45, 10–18.

[bib31] Nicodemus, F.E., Richmond, J.C., Hsia, J.J., Ginsburg, I.W., & Limperis, T. (1977). Geometrical considerations and nomenclature for reflectance*.* *NBS Monograph* 160, 1–52

[bib32] Nordvik, E., Schütte, S., & Broman, N. O. (2009). People's perceptions of the visual appearance of wood flooring: A kansei engineering approach. *Forest Products Journal,* 59(11-12), 67–74.

[bib33] Paulun, V. C., Schmidt, F., van Assen, J. J. R., & Fleming, R. W. (2017). Shape, motion, and optical cues to stiffness of elastic objects*.* *Journal of Vision**,* 17(1), 20–20. 10.1167/17.1.20 28114494

[bib34] Portilla, J., & Simoncelli, E. P. (2000). A parametric texture model based on joint statistics of complex wavelet coefficients. *International Journal of Computer Vision,* 40, 49–70.

[bib35] Rao, A. R., & Lohse, G. L. (1996). Towards a texture naming system: Identifying relevant dimensions of texture. *Vision Research,* 36(11), 1649–1669. 8759466 10.1016/0042-6989(95)00202-2

[bib36] Schmidt, F., Hebart, M. N., & Fleming, R. W. (2022). Core dimensions of human material perception. PsyArXiv, doi:10.31234/osf.io/jz8ks PMC11912425 40042912

[bib37] Sharan, L., Liu, C., Rosenholtz, R., & Adelson, E. H. (2013). Recognizing materials using perceptually inspired features. *International Journal of Computer Vision**,* 103, 348–371. 23914070 10.1007/s11263-013-0609-0 PMC3728085

[bib38] Sharan, L., Rosenholtz, R., & Adelson, E. (2009). Material perception: What can you see in a brief glance?*.* *Journal of Vision**,* 9(8), 784–784.

[bib39] Sharan, L., Rosenholtz, R., & Adelson, E. H. (2014). Accuracy and speed of material categorization in real-world images. *Journal of Vision**,* 14(9), 12–12. 10.1167/14.9.12 PMC4132332 25122216

[bib40] Tamura, H., Mori, S., & Yamawaki, T. (1978). Textural features corresponding to visual perception. *IEEE Transactions on Systems, Man, and Cybernetics,* 8(6), 460–473.

[bib41] Tanaka, M., & Horiuchi, T. (2015). Investigating perceptual qualities of static surface appearance using real materials and displayed images. *Vision Research,* 115, 246–258. 25536466 10.1016/j.visres.2014.11.016

[bib42] Van Assen, J. J. R., Barla, P., & Fleming, R. W. (2018). Visual features in the perception of liquids. *Current Biology**,* 28(3), 452–458. 29395924 10.1016/j.cub.2017.12.037 PMC5807092

[bib43] Wan, Q., Li, X., Zhang, Y., Song, S., & Ke, Q. (2021). Visual perception of different wood surfaces: An event-related potentials study. *Annals of Forest Science,* 78, 1–18.

[bib44] Wendt, G., Faul, F., & Mausfeld, R. (2008). Highlight disparity contributes to the authenticity and strength of perceived glossiness*.* *Journal of Vision**,* 8(1), 14–14. 10.1167/8.1.14 18318617

[bib45] Wendt, G., Faul, F., Ekroll, V., & Mausfeld, R. (2010). Disparity, motion, and color information improve gloss constancy performance. *Journal of Vision**,* 10(9), 7–7. 10.1167/10.9.7 20884605

[bib46] Wiebel, C. B., Valsecchi, M., & Gegenfurtner, K. R. (2013). The speed and accuracy of material recognition in natural images. *Attention, Perception, & Psychophysics**,* 75, 954–966. 10.3758/s13414-013-0436-y 23456971

