# Supplementary material for: Perceptual dimensions of wood materials
Source: J Vis. 2024 May 24;24(5):12. doi: 10.1167/jov.24.5.12 (PMC11129719; doi:10.1167/jov.24.5.12)
Supplement: Supplement 7 [file jovi-24-5-12_s007.docx]

**Perceptual Dimensions of Wood Materials**

**Supplementary Material**

Jiří Filip ^A^, Jiří Lukavský ^B^, Filip Děchtěrenko ^B^, Filipp Schmidt ^C^, Roland W. Fleming ^C^

1. The Czech Academy of Sciences, Institute of Information Theory and Automation, Pod Vodárenskou věží 4, 18200 Praha 8
2. The Czech Academy of Sciences, Institute of Psychology, Pod Vodárenskou věží 4, 18200 Praha 8
3. 1. Experimental Psychology, Justus Liebig University of Giessen Germany, 2. Centre for Mind, Brain and Behaviour, Universities of Marburg, Giessen and Darmstadt, Otto-Behaghel-Str 10, 35394 Giessen, Germany
4. **Similarity matrices computation**

**Computation of similarity matrix from similarity embedding**

Similarly to Hebart, Zheng, Pereira, & Baker, 2020, we defined object similarity in the triplet 2AFC task as the probability *p(i,j)* of participants choosing samples *i* and *j* to be visually closer than samples *i* and *k*. Therefore, to compute similarity from the learned embedding for all samples *n*_s_=30, we created all predicted choices for all possible triplets and calculated the mean choice probability for embedding values x for each pair of samples:


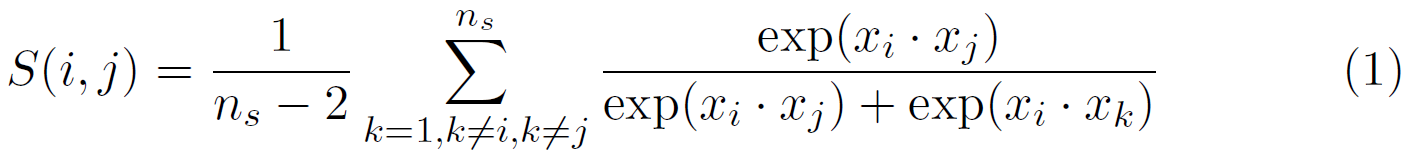


**Computation of similarity matrix from mean rating values**

We compute similarity of two samples for attribute *a* given known rating values R for all samples (rows) and attributes (columns) using the following equation:


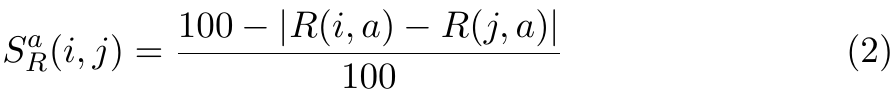


A joint similarity matrix combining all rating contribution of all ten attributes is computed by is the root mean square individual-attributes similarity matrices:


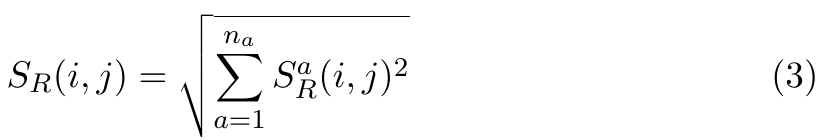


1. **VICE algorithm training**

We tested the VICE model on 76 different combinations of input parameters such as et, spike, slab, pi and distribution (Gaussian, Laplace) (cf. Muttenthaler, Zheng, McClure, Vandermeulen, Hebart, & Pereira, 2022). Results are shown in Fig. S1(a), where the tested models are rank ordered according to test accuracy (red), with the corresponding training accuracy (blue). The converged models are highlighted as circles. Fig. S1(b) shows that the number of dimensions is relatively stable, within a range between 5 to 14 and a typical value of 10 dimensions.


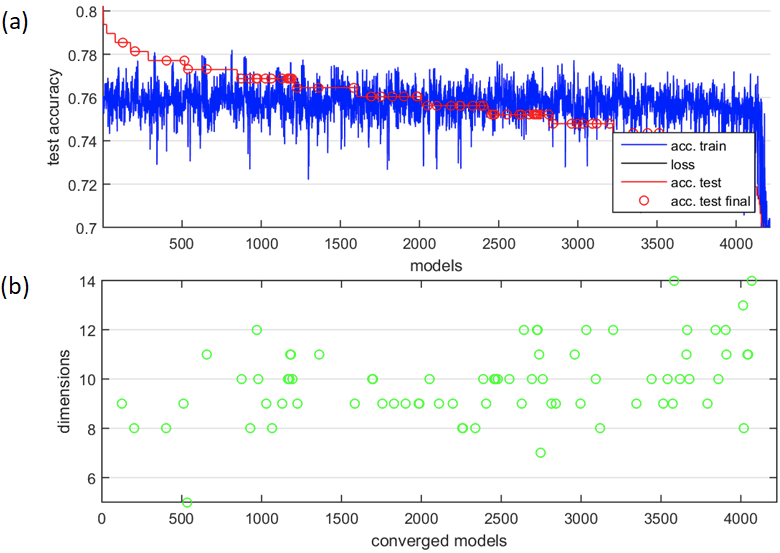


**Fig. S1** Results of grid search across different VICE model parameters. (a) Model accuracies on train (blue) and test (red) sets (across all tested models) sorted according to accuracy on test set (red), and (b) corresponding obtained numbers of dimensions for converged models (also denoted as circles in (a)).

Fig. S2 shows the training process of the best performing converged model with the highest accuracy.

**
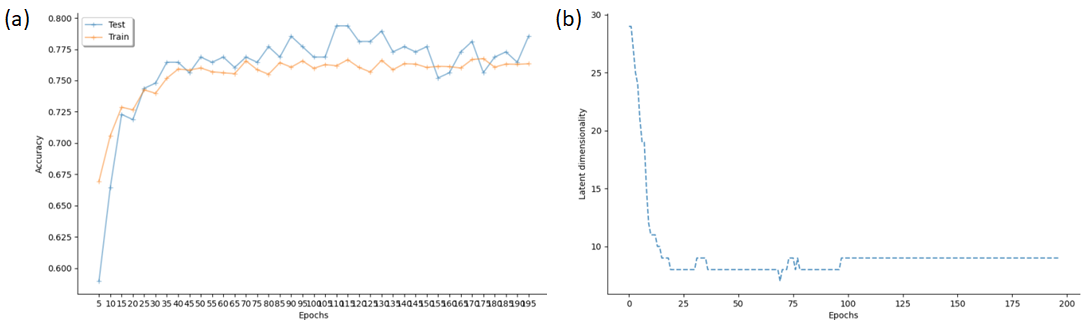
**

**Fig. S2** Training process of the best performing model. (a) Model accuracy on the training (blue) and test (orange) dataset, (b) dimensionality reduction over 200 epochs of VICE algorithm.

1. **Louvain community detection**

We also applied the community detection method (Blondel, Guillaume, Lambiotte, & Lefebvre, 2008) on the estimated similarity matrix. The resulting three clusters visualised in Fig. S3 can be interpreted as (1) contrast/roughness, (2) non-directional/low frequency, (3) directional/high frequency modes. These results are in agreement with the results of hierarchical clustering and MDS analysis.


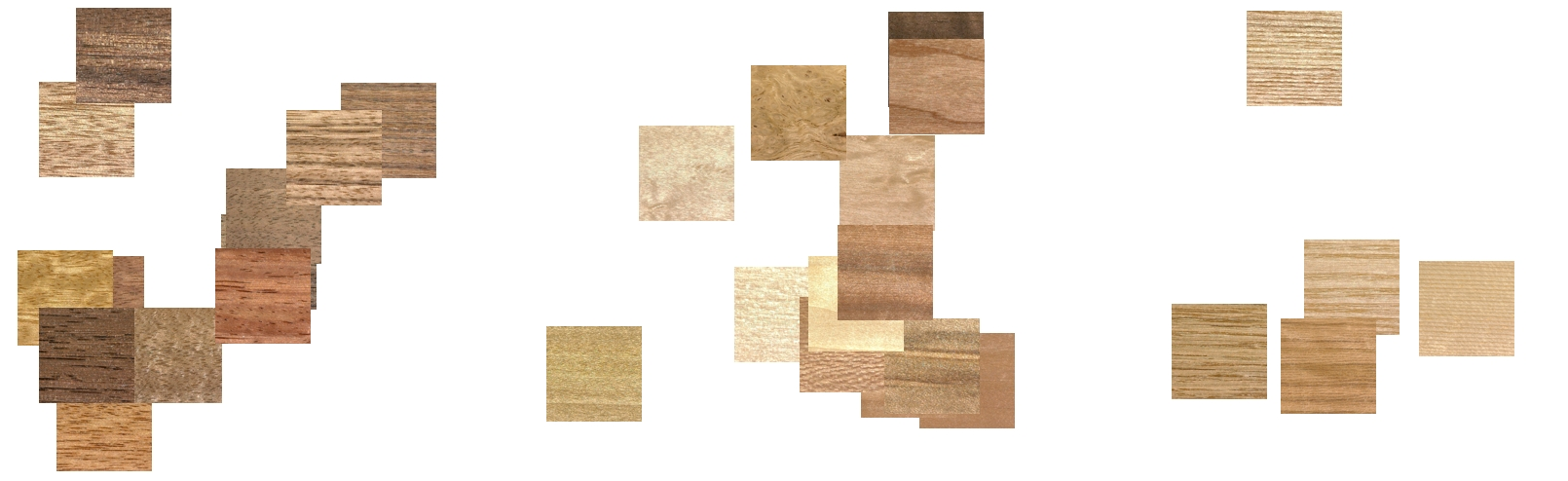


**Fig. S3** Clustering based on the Louvain community detection method divided the material samples into three clusters. See supplementary video [movie_Louvain.avi].

1. **Similarity judgement data dimensionality analysis**

As the dimensionality of our dataset is unknown, we follow a recent approach by (Künstle, von Luxburg, & Wichmann, 2022) to estimate the number of perceived dimensions from triplet experiments, based on triplet embedding accuracy. When splitting our triplet dataset into 90% training and 10% test samples, we obtain an ordinal Euclidean embedding (Haghiri, Wichmann, & von Luxburg, 2020) for the perceptual ratings. This procedure always leads to a decreasing triplet error (cross-validated on the validation set) with an increasing number of dimensions until a sufficient number of dimensions has been reached. We ran a cross-validation with 10 repetitions, resulting in a drop of accuracy with more than 6 dimensions. This suggests that inherent dimensionality of our dataset is close to 6 perceptual dimensions. Note that our analysis shown in Fig. S1(b) reports a dimensionality of the typical estimated similarity embedding between 8 and 10 dimensions. This seems to contradict the estimate of the inherent dimensionality of 6 as reported above (and shown in Fig. S4). However, our linear regression analysis (blue bars in Fig. 12(a)) suggests that several of our similarity dimensions (namely dimension 7) cannot be reliably predicted from the appearance ratings, which might suggest that: (1) our rating dimensions do lack some important visual features, or (2) the number of representational dimensions is lower than the estimate of the VICE algorithm. In favour of the latter, the factor loadings of individual dimensions (Fig. 3(b)) show a drop in loadings for dimensions higher than 5. Also, when using PCA on the rating data to test whether intercorrelations (Fig. 8(b)) allow us to reduce the dimensionality, we end up with not more than 6 dimensions.


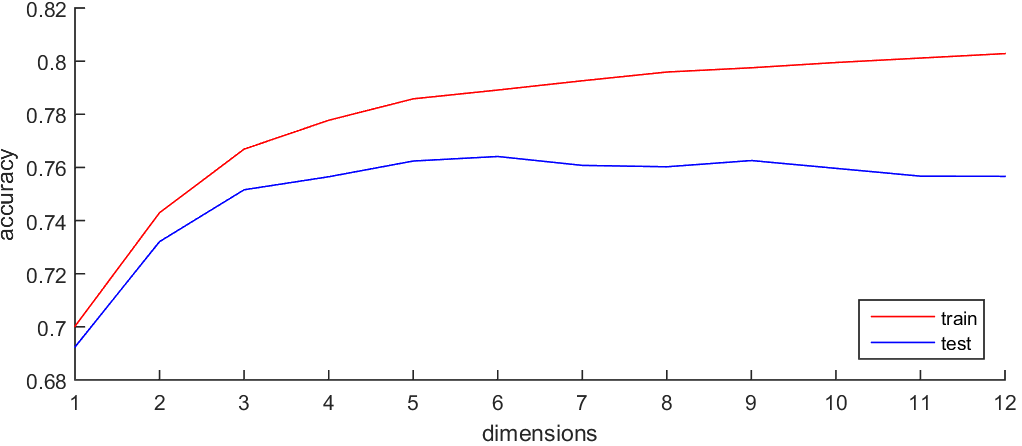


**Fig. S4** Triplet ordinal embedding error as a function of the number of dimensions for training and test set of triplets from our similarity experiment.

To demonstrate contributions of individual dimensions, we computed the accuracy of our prediction on the training and test data sets for a variable number of dimensions in embedding (sorted by the total loading across all materials) and obtained the graphs in Fig. S5, which demonstrates that adding more dimensions than seven did not improve model prediction accuracy on test data significantly. The graphs show similar performance on the best five embeddings E1-E5.

Train data Test data


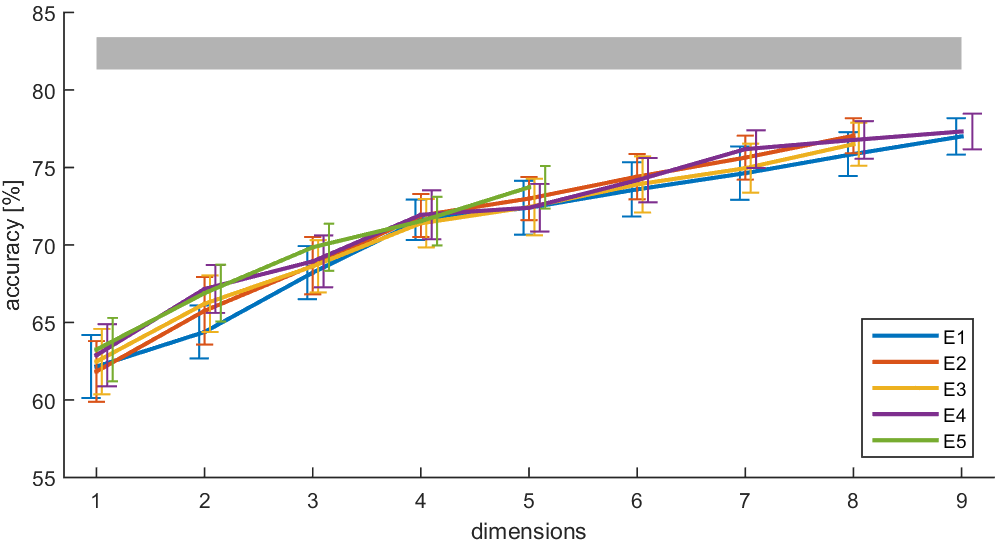

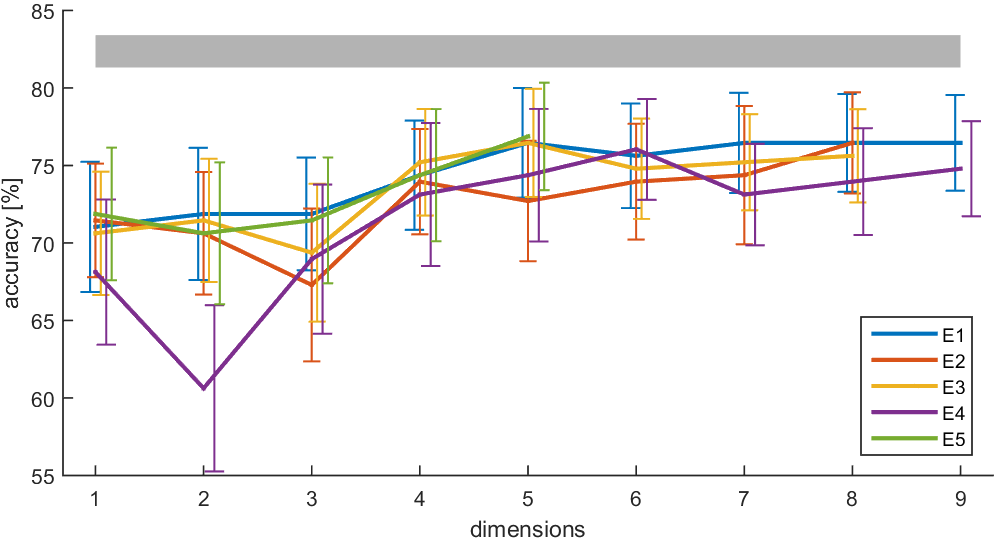


**Fig. S5** Accuracy of VICE model prediction as a function of effective number of dimensions on train and test datasets. This is demonstrated on the five best embeddings E1-E5.

1. **Stability of similarity embeddings**

To show model estimation stability across different hyperparameters and dimensions we show embeddings obtained from 5 best models and its parameters below:

| **E1: 9 dims**  epochs 197  LR 0.0005  Laplace  Spike 0.125  Slab 0.2  Pi 0.4 | **E2: 8 dims**  epochs 264  LR 0.0005  Gaussian  Spike 0.125  Slab 0.2  Pi 0.6 | **E3: 8 dims**  epochs 207  LR 0.0005  Laplace  Spike 0.125  Slab 0.2  Pi 0.5 | **E4: 9 dims**  epochs 340  LR 0.002  Laplace  Spike 0.75  Slab 0.2  Pi 0.4 | **E5: 5 dims**  epochs 121  LR 0.0005  Gaussian  Spike 0.125  Slab 0.2  Pi 0.4 |
| --- | --- | --- | --- | --- |


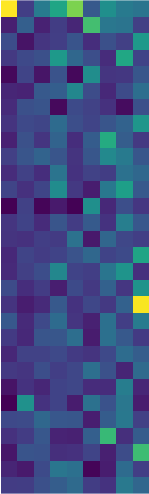

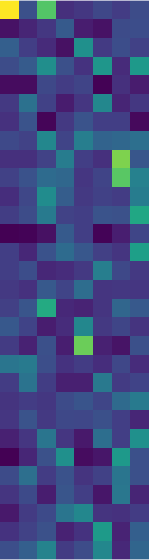

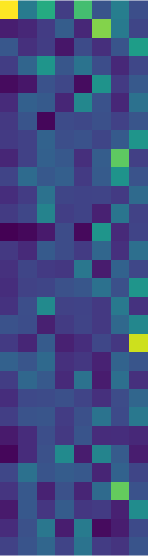

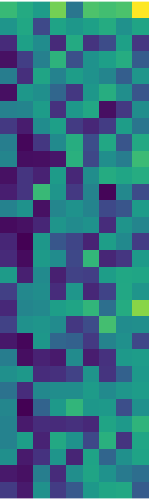

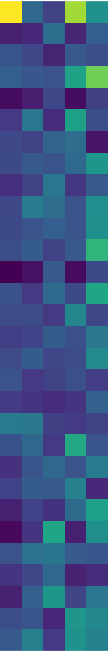


**Fig. S6** Five embeddings having best results on test dataset.

We clearly observe that different hyper-parameters produce similar embeddings, as verified by computing similarity matrices for each embedding shown in Fig. S7. Note, that although the embedding E4 was obtained by quite a different set of parameters and it also looks different from the remaining ones, its similarity matrix is still very similar to those for the other embeddings. The matrices are very similar. We computed the correlation of the first matrix (the best) to the remaining ones. The correlation was computed without diagonal elements and their mean value is 0.922.


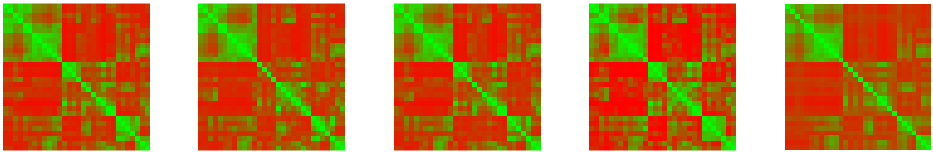
 Correlations: E1-E2 0.905 E1-E3 0.965 E1-E4 0.935 E1-E5 0.885

**Fig. S7** Similarity matrices of the five best embeddings.

1. **Rating experiment analysis**

The interface of the rating experiment is shown in Fig. S8.


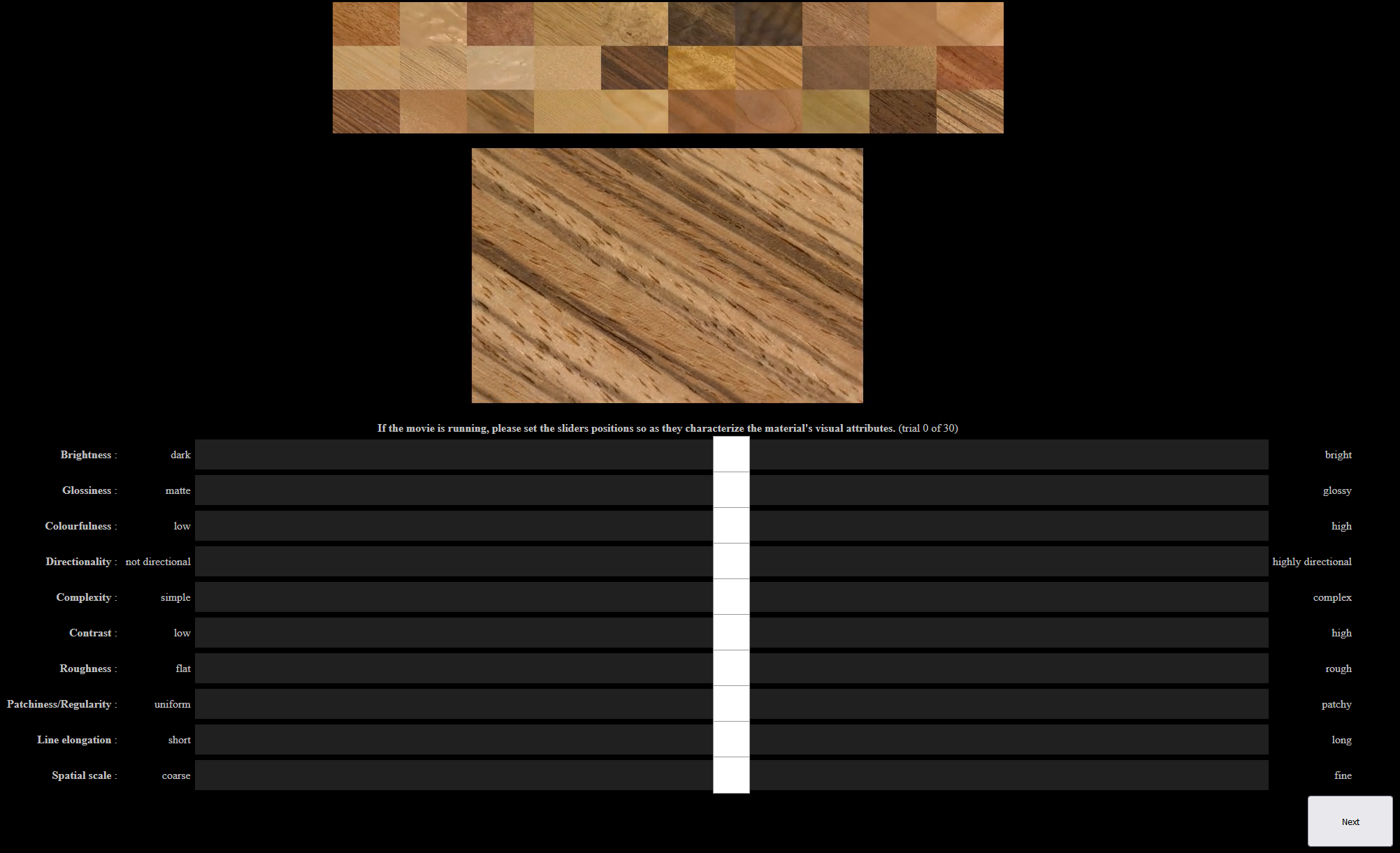


**Fig. S8** Example stimulus frame from the rating experiment.

Instructions of the rating experiment were as follows: “*Below the video, there are 10 sliders for the visual material attributes. Your task is to adjust the slider position for each material. You may consider the appearance of the other materials (at the top of the screen) to choose your rating appropriately within the range we are testing*.”

The following visual attributes are evaluated:

1. **Brightness** - how bright is the material?

2. **Glossiness** - how shiny is the material?

3. **Colourfulness** - how colourful is the material?

4. **Directionality** - presence of directional structures in the texture

5. **Complexity** - how complex are the patterns on the surface?

6. **Contrast** - difference in brightness of surfaces patterns

7. **Roughness** - smoothness of surface profile, range of surface heights

8. **Patchiness/Regularity** - how uniform is the pattern?

9. **Line elongation** - are line elements shorter dashes or extended lines?

10. **Spatial scale** - are patterns large and broad, or small and fine?

1. **Rating results analysis**

Mean values of participant responses for each resting attribute, and normalised distribution of all pooled ratings is shown in Fig. S9.


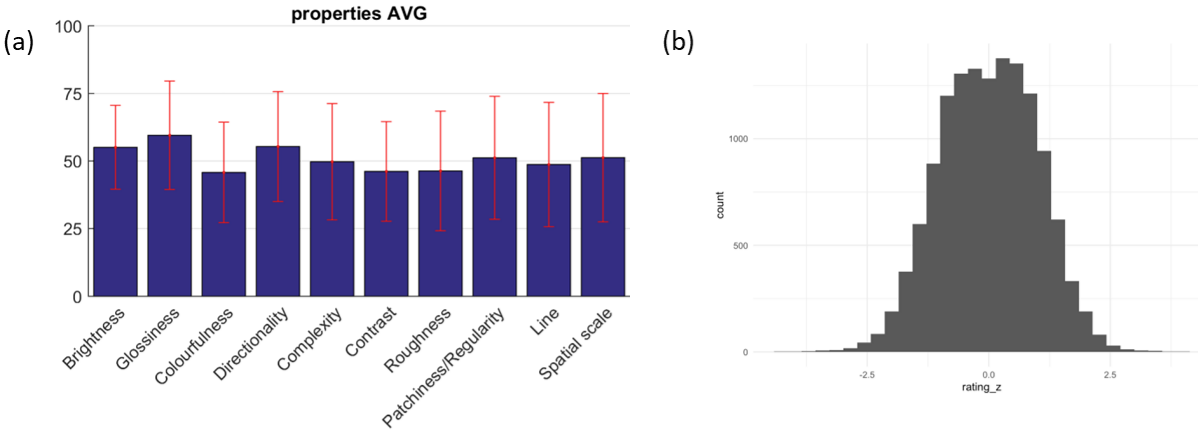


**Fig. S9** Rating data analysis. (a) Mean values of participant responses across all materials with SD values, and (b) normalised distribution of all pooled ratings.

To evaluate the consistency between participants, we correlate the ratings of each participant within a scale to the corresponding mean rating (Fig. S10). Although overall correlations are pretty high, there is a heavy tail towards zero and even some negative correlations. Also, the consistency between participants varies between rating dimensions, for example, with more consistent judgements for brightness (stronger correlations and less variability). Tab. S1 shows intra-class correlations for individual rating dimensions.

**
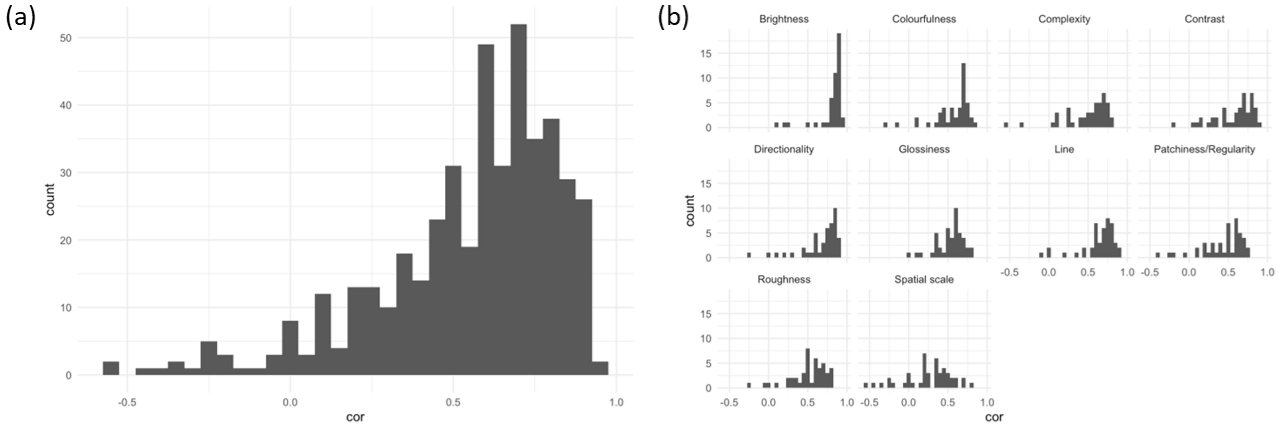
**

**Fig. S10** Correlations between individual ratings of participants to the mean ratings, within each rating dimension. (a) Results across all attributes and (b) within individual dimensions.

**Tab.S1** Intra-class correlations for individual rating dimensions.

| **rating dimension** | **single random raters** | **average**  **random**  **raters** |
| --- | --- | --- |
| brightness | 0.618 | 0.986 |
| glossiness | 0.219 | 0.927 |
| colourfulness | 0.262 | 0.941 |
| directionality | 0.416 | 0.970 |
| complexity | 0.197 | 0.917 |
| contrast | 0.301 | 0.951 |
| roughness | 0.220 | 0.927 |
| patchiness/regularity | 0.164 | 0.898 |
| line | 0.386 | 0.966 |
| spatial scale | 0.041 | 0.659 |

1. **Samples alignment along the first three MDS dimensions**

The MDS analysis distributed our 30 samples to three dimensional space. Distribution of samples along these dimensions is shown in Fig. S11 in a form of mappings of wood sample locations in the MDS space. This mapping is done after Procrustes alignment, based on the results from similarity judgments. The judgments (in red colour) relate to: (a) rating attributes, represented by the L2 norm of all attributes, (b) linear regression of similarity matrices, and (c) the L2 norm of image statistics - all shown in blue colour.

**
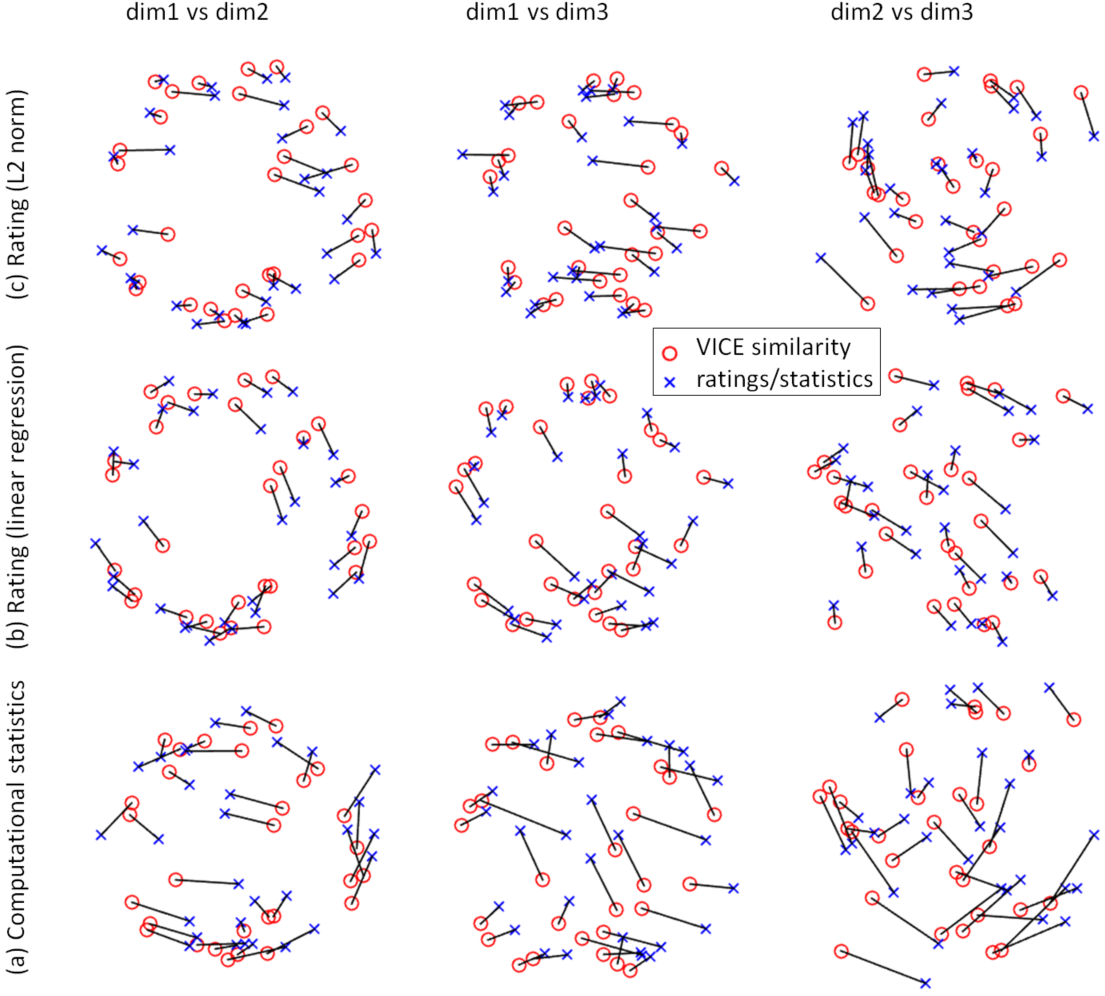
**

**Fig. S11** Procrustes alignment of MDS dimensions computed from similarity matrices. (red) VICE similarity model MDS, (blue) rating attributes MDS for similarity matrix obtained by L2- norm of attributes (a) linear regression of similarity matrices (b), and computational statistics MDS (c).

1. **Visualisation of linear regression coefficients**

In order to analyse the relationship between rating attributes and VICE dimensions, we performed linear regression in both directions. FIg S12 demonstrates contribution of individual similarity dimensions/rating attributes by means of regression coefficients for different rating attributes / VICE dimensions.

**
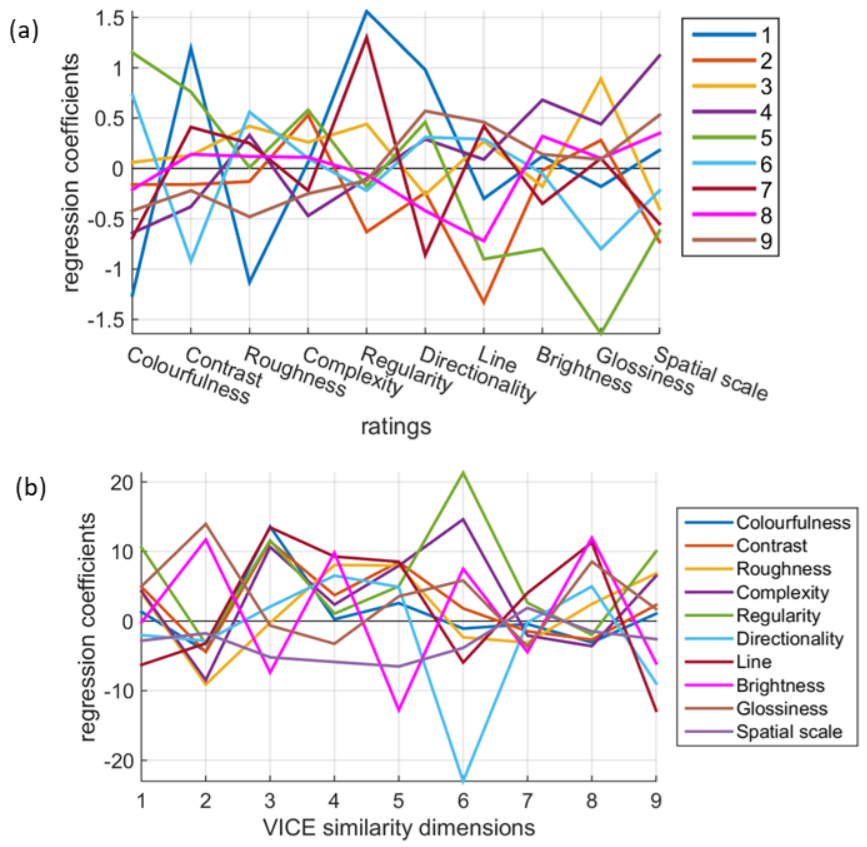
**

**Fig. S12** Values of regression coefficients of rating attributes (a) and similarity dimensions (b) showing contribution of individual rating attributes/VCE similarity dimensions for reconstruction of each similarity dimension/rating attribute.

Similarly, Fig. S13 shows, in the form of regression coefficients, a contribution of computational image statistics for linear regression of VICE similarity dimensions.


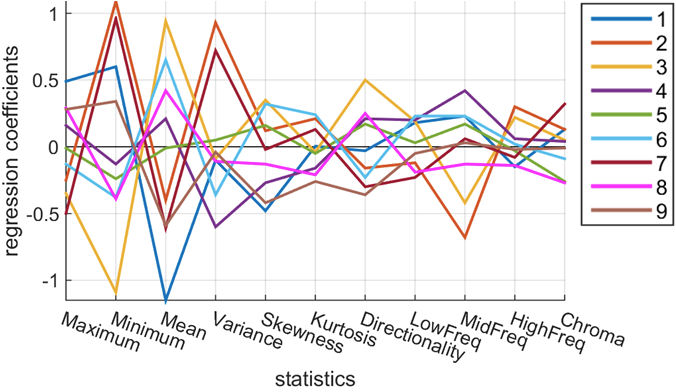


**Fig. S13** Normalised regression coefficients showing contribution of computational statistics for reconstruction of each VICE similarity dimension.

**A list of supplementary movies**

1. [movie_samples_stimuli.avi] - 30 test wood video sequences used in the experiments
2. [movie_similarity_vs_rating.avi] - rank ordered samples (left) according to loadings values of similarity dimensions, (right) mean rating attributes (the five closes and 5 the most distant)
3. [movie_MDS_simmat_linreg.avi] - distribution of samples along three MDS dimensions (top) for similarity judgements, (bottom) for rating study
4. [movie_MDS_simmat_stat.avi] - distribution of samples along three MDS dimensions (top) for similarity judgements, (bottom) for computational statistics obtained from image sequence.
5. [movie_similarity_scaled.avi] - rank ordered samples scaled according to loadings values of similarity dimensions
6. [movie_Louvain.avi] - result of community detection using Louvain method (computed from similarity matrices), distributing samples to three clusters for (top) similarity judgements and (bottom) rating study.
